# Sleep as a potential link between exposome, mental health and cognitive development in children and adolescents - a scoping review

**DOI:** 10.1186/s13690-026-02016-9

**Published:** 2026-07-17

**Authors:** Natalia Vincens, Kerstin Persson Waye, Michael G. Smith, Peter Lercher, Angel M. Dzhambov, Maria Klatte, Larisa Leist, Thomas Lachmann, Jan Spilski, Dirk Schreckenberg, Christin Belke, Gordana Ristovska, Sonja Jeram, Katja Kanninen, Arto Alatalo, Izaque de Sousa Maciel, Dick Botteldooren, Timothy Van Renterghem, Jenny Selander, Arzu Arat, Kim White, Maria Foraster, Jordi Julvez, Mai Quynh Nguyen, Charlotte Clark, Irene van Kamp

**Affiliations:** 1 Sound Environment and HealthSchool of Public Health and Community Medicine, Institute of Medicine, University of Gothenburg, Box 414, Gothenburg, SE-405 30 Sweden; 2 Institute of Highway Engineering and Transport Planning, Graz University of Technology, Graz, Austria; 3 Environmental Health Division, Research Institute at Medical University of Plovdiv, Medical University of Plovdiv, Plovdiv, Bulgaria; 4 RPTU, Rheinland-Pfälzische Technische Universität, Kaiserslautern, Germany; 5 Centre for Applied Psychology, Environment and Social Research (ZEUS GmbH), Hagen, Germany; 6 Institute of Public Health of the Republic of North Macedonia, Faculty of Medicine, Ss. Cyril and Methodius University in Skopje, Skopje, North Macedonia; 7 National Institute of Public Health (NIJZ), Ljubljana, Slovenia; 8 A.I. Virtanen Institute for Molecular Sciences, University of Eastern Finland, Kuopio, Finland; 9 Department of Environmental and Biological Sciences, University of Eastern Finland, Kuopio, Finland; 10 Ghent University, Ghent, Belgium; 11 Unit of Occupational Medicine, Institute of Environmental Medicine, Karolinska Institutet, Stockholm, Sweden; 12 National Institute for Public Health and the Environment, Bilthoven, Netherlands; 13 ISGlobal – Barcelona Institute for Global Health, Barcelona, Spain; 14 Clinical and Epidemiological Neuroscience Group (NeuroEpia), Institute of Biomedical Research of Southern Catalonia (IRB CatSud), Bercelona, Spain; 15 Population Health Research Institute, St George’s University of London, London, UK

**Keywords:** Exposome, Sleep, Mental health, Cognition, Children and adolescents, mediation or moderation

## Abstract

**Background:**

Children’s and adolescents’ mental health and cognitive development are shaped by complex environmental exposures across their life-course. The exposome framework provides an integrative approach to assess the interlinkages. While sleep is a prerequisite for mental and cognitive health, its role as an underlying pathway remains unclear. This review aims to map and contextualize the evidence on whether sleep mediates or moderates associations between the exposome and mental health, well-being, or cognitive outcomes in individuals aged 0–21 years.

**Methods:**

Following PRISMA-ScR guidelines, we conducted a scoping review of peer-reviewed studies published in English from 2000 to January 2025. Scopus and PsycINFO were searched, supplemented by reference screening and cohort-based searches. Two reviewers independently screened records and charted data, which were synthesised narratively.

**Results:**

Twelve studies examined the full causal chain from multiple environmental exposures to mental health or cognitive outcomes via sleep, with 34 additional studies addressing specific partial pathways. Most studies were cross-sectional and conducted in high-income countries. Sleep was primarily assessed using self- or parent-reported measures. Evidence suggested that sleep may mediate or moderate associations between the exposome and mental health or cognitive outcomes, however, longitudinal evidence remains notably limited. The diversity of exposures and domains points to the effect being a result of multiple exposures emphasising the importance of an exposome perspective within early life exposures and outcomes.

**Conclusions:**

Sleep represents an important pathway linking the exposome to mental health and cognitive development, however longitudinal, child-centred exposome research remains scarce.

**Supplementary Information:**

The online version contains supplementary material available at 10.1186/s13690-026-02016-9.

**Table:** 

Text box 1. Contributions to the literature
This scoping review brings together evidence on how children’s environments (home, school, and neighbourhood) may affect mental health and learning through sleep, a pathway not sufficiently examined across studies.
Shows that poor sleep may be an important link between multiple environmental exposures and mental health, highlighting sleep as a key target for public health action.
Identifies major gaps in current research, including a lack of long-term studies and limited focus on children’s real-life combined exposures.
Supports the need for policies and interventions that improve sleep conditions in children’s daily environments to promote better mental health and development.

## Background

Mental disorders are prevalent worldwide and constitute the second largest cause for long-term disability. Globally, about one in seven individuals aged 10–19 years old is affected, accounting for 15% of the global burden of disease in this age group. Reports from high-income countries indicate a marked increase over the last 10–15 years [1, 2]. In children and adolescents, these disorders may manifest as challenges in emotional and cognitive regulation and social functioning, potentially interfering with identity development, peer relationships, and school performance [2]. Furthermore, mental disorders emerging in childhood and adolescence are associated with adverse outcomes that may extend into adulthood, including impaired academic attainment, reduced quality of life, and long-term social and economic consequences [3–7].

Mental health and cognitive functioning result from a complex interplay of genetic, psychological, physical and social factors in the environment [8–12]. The complexity of environments calls for an integrated perspective in which salutogenic and pathogenic exposures as well as their timing, frequency and accumulation in a developmental perspective are essential elements in isolation and combination [13]. Furthermore, bidirectional relationships between outcomes and environmental factors are possible, highlighting the importance of longitudinal study designs for disentangling causal pathways and reducing bias in cause-and-effect inference [9].

Sleep plays a crucial role in maintaining psychological and physical health. From studies on adults, it is well known that undisturbed sleep regulates blood pressure, the immune function [14], endocrine activity, glucose metabolism [15], as well as cognitive processes like memory and alertness [16, 17]. While these mechanisms are likely to be relevant for children and adolescents [18], empirical evidence directly examining the physiological consequences of sleep disruption in younger populations remains limited [19]. This gap is notable given that children’s sleep undergoes substantial changes across developmental stages, potentially modifying both vulnerability and resilience to sleep disturbances. In early life, children experience longer sleep durations and a higher proportion of slow-wave sleep and rapid eye movement (REM) sleep, which gradually decline with age [20]. Consensus guidelines recommend age-specific sleep durations to support optimal health ranging from 12 to 16 h for infants (4–12 months), to 8–10 h for adolescents (13–18 years) [21].

Sleep duration is positively associated with cognitive performance, and particularly with executive functioning, and internalising and externalising behaviours. In contrast to adults, sleep duration in children appear though to be less associated with sustained attention and memory [22]. Beyond sleep quantity, undisturbed sleep or sleep quality is necessary for recovery, daytime alertness, attention, mood and emotion regulation, as well as academic performance [23–26].

During adolescence, vulnerability may increase due to social and emotional demands, including nighttime attention to social media. Together with the circadian rhythm change, characteristic to this period [27], social and emotional demands may substantially compromise sleep quality and quantity [28]. In the short term, sleep loss may affect mood, memory and learning [29], but if chronic, such disturbances may be linked to higher scores on suicidal ratings [21], depression and impaired school achievement [30–32]. While some of these studies may not fully account for the bidirectionality of cause and effect, evidence from longitudinal studies shows that short sleep duration predicts depressive symptoms and disorders [32–34].

Adolescence is also a period of intense white-matter development in prefrontal regions [28]. This concerns regions of vital importance for problem-solving, coping, interpersonal skills, self-regulation, and emotional control and includes the myelination of axons pathways connecting amygdala in the limbic system with the orbitofrontal and medial prefrontal cortices. Studies indicate that sufficient sleep is needed for myelination [35] and functional magnetic resonance imaging studies confirm that poor sleep among adolescents was associated with heightened affect and impulsivity among those with low functional connectivity [36, 37]. In addition, sleep deprivation was associated with greater connectivity between amygdala and brain stem regions involved in autonomic regulation. Poor sleep may thus not only decrease the ability for self-regulation and emotional control but also amplify acute stress responses [28].

Taken together, the growing child and adolescent can thus be expected to be particularly vulnerable for social and physical exposures during their development [38, 39] however research on how the totality of exposures in a life-course perspective -the exposome - affects adolescents’ or children’s sleep, mental health and cognitive development is very limited. The exposome concept, first introduced by Wild [40, 41] as a complement and response to the genome initiative, highlight the need to consider the internal and external exposures for health. The internal exposome usually refer to internal processes e.g., metabolism, endogenous hormones, gut microflora, inflammation, oxidative stress, while the external exposome can be subdivided into the physical (e.g. indoor and outdoor air pollutants, noise, light, natural and built environment) and social (e.g. social capital, education, financial status, social, and psychosocial factors at the individual and community levels). Given the complexity and timing of exposures, and age-related vulnerability, an exposome approach provides opportunities to consider how children’s and adolescents’ development and mental health are affected by the complex interplay of external and internal exposures over the life-course and furthermore how important mediators such as sleep may mediate or moderate these associations. Vulnerability is here referred to being exposed to more frequent or higher levels, being more susceptible and/or having lower capacity to deal with a given exposure and or effect [42]. Following the framework proposed by Persson Waye et al. [13], all three aspects of vulnerability are related to the biopsychosocial development of the child and adolescent period.

Given these insights there are surprisingly few exposome studies within mental health on children and adolescence [43]. Until recently, research on exposome and children’s mental health and cognition has furthermore largely focused on chemical, biological, and physical exposures, or on social factors separately [44–47].

The EU-funded project Early Environmental quality and life-course mental health effects (Equal-Life) adopts the exposome framework to elucidate salutogenic and pathogenic determinants for children’s development and mental health [48]. To further alleviate interventions, Equal-Life undertook reviews of the literature on how the exposome via selected potential mediators (stress/restoration, sleep, and coping/self-regulation) may affect mental health (including well-being and quality of life constructs) and cognition in a life-course perspective over the age period of 0–21 years.

Against this background, the present review aims to map and contextualize existing evidence from an exposome perspective, conceptualizing the totality of physical and social exposures through the lens of children’s vulnerability across different developmental stages. Whereas prior exposome research has primarily focused on organic and non-organic chemical compounds, comparatively little attention has been paid to sleep as a potential pathway linking environmental and social exposures to children’s and adolescents’ mental health and cognitive development. This review therefore seeks to address an important scientific gap by examining the role of sleep as a mediating mechanism through which physical, internal, and social exposures may influence developmental outcomes.

### Aims

We aimed to systematically search, summarize, and critically discuss how sleep may mediate or moderate the effect of exposome on mental health and /or cognitive development within the age group 0–21 years. In addition, we explored how partial evidence for exposome and sleep, respective sleep and mental health/cognitive development could contribute to a more integrated understanding.

## Methods

### Scoping review

To identify studies examining the full pathway of exposome to mental health/cognitive development with Sleep as a mediator or moderator - a scoping review was adopted. A scoping review was selected due to the limited prior knowledge regarding the breadth and nature of the literature addressing the child exposome in relation to mental health and sleep. This approach enabled us to systematically explore and map the emerging body of evidence within this field. In addition, there was a need to clarify key concepts and definitions and to identify and examine existing knowledge gaps. Taken together, these considerations supported the adoption of a scoping review in place of a systematic review. In line with methodological guidance by Mak and Thomas [49], we also recognised the importance of assembling a multiprofessional research team. The team comprised expertise in Environmental and Social Medicine, Epidemiology, Psychology, Child Development, Public Health, technical exposure assessment, as well as professionals experienced in conducting scoping reviews and information specialists (librarians).

#### Information sources and search strategies

A review protocol was developed following PRISMA-ScR recommendations [50]. We performed literature searches in the following databases: Scopus and PsycINFO. The searches were conducted by a team of librarians at the University of Gothenburg: in June 2020, in September 2020 with revised search terms (adding a few new terms we found were missing in the first searches), in December 2022 and January 2025 for updates, using the revised search strategy. The searches based on titles and abstracts targeted peer-reviewed studies, published in English since 2000. The search strategy and search terms used in the latest searches are shown in Table S1, Supplement S1 and S2. Applying similar search parameters (i.e., conceptualization on exposome, mediators, outcomes, as well as language, time span), we also searched papers based on the cohorts and school studies included in the European exposome project Equal-Life (https://www.equal-life.eu/en), and the reference lists of relevant review papers and articles.

#### Eligibility criteria

We employed strict criteria for studies to be included in the review see Table 1. Study records had to be available in English, study the association between the exposome and child/adolescent mental health/cognition, and investigate the role of sleep as a relevant mechanistic factor. Empirical studies of observational or experimental design were eligible, but reviews and ecological studies were excluded.

**Table 1:** Inclusion and exclusion criteria used to screen study records in the scoping review

	Inclusion criteria	Exclusion criteria
Population	Humans, children/adolescents (0–21 years) or at least > 50% of participants ≤ 21 years old	Animal studies, < 50% of participants < 21 years old, pregnant women
Study design	Cross-sectional, case-control, cohort, case crossover, (quasi-) experimental, randomized controlled trial	Reviews, ecological/area-level studies
Exposures	Multiple physical exposures or at least two exposures pertaining to different exposome domains, one of which to the physical exposome; objectively measured and/or self-reported	Single exposures or multiple exposures from only social or internal exposome domain
Outcomes	Child/adolescent mental health and/or cognition and/or well-being/quality of life; objectively measured or self-reported (different than above; mental health, positive or negative affect, externalizing or internalizing symptoms, cognitive functioning, and academic performance.	Only maternal outcomes, perceived general health, physical health outcomes
Mediator/moderator	Sleep as potential mechanism, subjective or objective measured	Sleep only treated as a confounder, but not as an intermediate variable

#### Screening, selection, and data charting

We used the web-tool Rayyan to support the initial screening phase, based on title and abstract only. Two reviewers independently screened abstracts and, if needed, full manuscripts for eligibility. Differences between reviewers’ assessments were discussed until consensus was reached or by a third reviewer. The log of all excluded studies and the reasons for their exclusion were recorded. Reviewers extracted the following data from each included paper: study design, location and period, sampling strategy and sample size, exposures category and assessment, outcome definition and assessment, mediator definition and assessment, statistical analysis, and adjustments (e.g. confounders). To do this we used a pre-designed data extraction form.

### Supporting mechanistic evidence by partial pathways

To explore additional evidence, we included studies on the partial pathways: Exposome to sleep and sleep to mental health and cognitive development. The process is illustrated in Figure S1. Evidence of the partial pathways of exposome to sleep and sleep to outcomes were explored including articles identified at the screening for the scoping review, that did not fulfil the inclusion criteria for the full pathways For example, if a study only included one exposure, it would be excluded from the scoping review, but could provide useful information for understanding mechanisms. To be included these articles had to fulfil the criteria for the respective pathways. In addition, we reviewed studies from other sources (Papers based on the cohorts and school studies included in Equal-Life, the reference list of relevant review papers and articles; as well as papers provided by expert knowledge within the Equal-Life advisors and team.

### Synthesis of finding

Study characteristics were described narratively. Included studies were also tabulated for the analysis. In synthesising the evidence, particular attention was paid to study design, exposure domains, sleep measures, outcomes, and analytical approaches, including mediation and moderation models. Where multiple publications were based on the same underlying cohort, this was noted during synthesis and findings were interpreted considering the shared study population and the differing research questions and analytical models, to avoid overemphasising evidence from a single dataset. In line with PRISMA-ScR guidance, no formal risk of bias or methodological quality assessment was undertaken, as the purpose of this scoping review was to map the scope, characteristics, and conceptual approaches of the available evidence rather than to appraise the strength of effect estimates. Where available, we also considered the adjustment strategies used in the studies and how covariates were conceptualized (e.g., co-exposure, moderators, confounders). Given the scoping nature of the review, we did not formally appraise risk of bias, but we used this information to contextualize the interpretation of reported associations.

Due to the heterogeneity of included studies in terms of populations, exposure definitions, sleep measures, outcome measures, and analytical approaches, no formal meta-analysis was conducted. We therefore applied a narrative synthesis approach, consistent with the scoping objective of mapping the field and identifying conceptual and methodological gaps.

### Definitions

For the search, retrieval, synthesis and analysis of the evidence, key concepts and operational definitions are summarised in Table 2.

**Table 2:** Key concepts and operational definitions used in this review

**Exposome**
The exposome was defined as the totality of environmental exposures across the life-course [40], adapted to a child-centred perspective that accounts for developmental timing, accumulation, and co-occurrence of exposures [13]. This framework includes physical, social, and internal exposure domains, and recognises that children’s exposures are shaped by the places they inhabit, the activities they engage in, and their social contexts. For study eligibility, research explicitly referring to the exposome or studies examining multiple or cumulative exposures were considered, with priority given to several physical exposures alone or in combination with social or internal exposures.
**Sleep**
Sleep was defined using objective and subjective assessment approaches commonly applied in epidemiological research. Instrumental measures included polysomnography and actigraphy, while self-reported measures comprised validated questionnaires assessing sleep duration, sleep disturbances (external factors affecting sleep), sleep quality, poor sleep (e.g. difficulties falling asleep, wake-ups and having difficulties going back to sleep and feeling tired in the morning) and daytime sleepiness, including the Insomnia Severity Index [51], Karolinska Sleepiness Scale [52], and Epworth Sleepiness Scale [53]. Circadian-related biomarkers (e.g. cortisol, melatonin) were included.
**Mental health**,** wellbeing**,** and cognition**
Mental health outcomes were defined as psychological or psychiatric symptoms assessed using validated questionnaires or clinical diagnoses. Wellbeing was defined as positive psychological functioning in line with the World Health Organization definition (WHO, 2004), encompassing both hedonic and eudaimonic dimensions [54, 55]. Cognitive outcomes included executive functions, learning, language, and academic achievement assessed using validated neuropsychological or educational measures, such as the Child Behaviour Checklist, Strengths and Difficulties Questionnaire, McCarthy Scales of Children’s Abilities, and the NIH Toolbox ADDIN EN.CITE [56–58].

## Results

### Scoping review

**Fig. 1:**
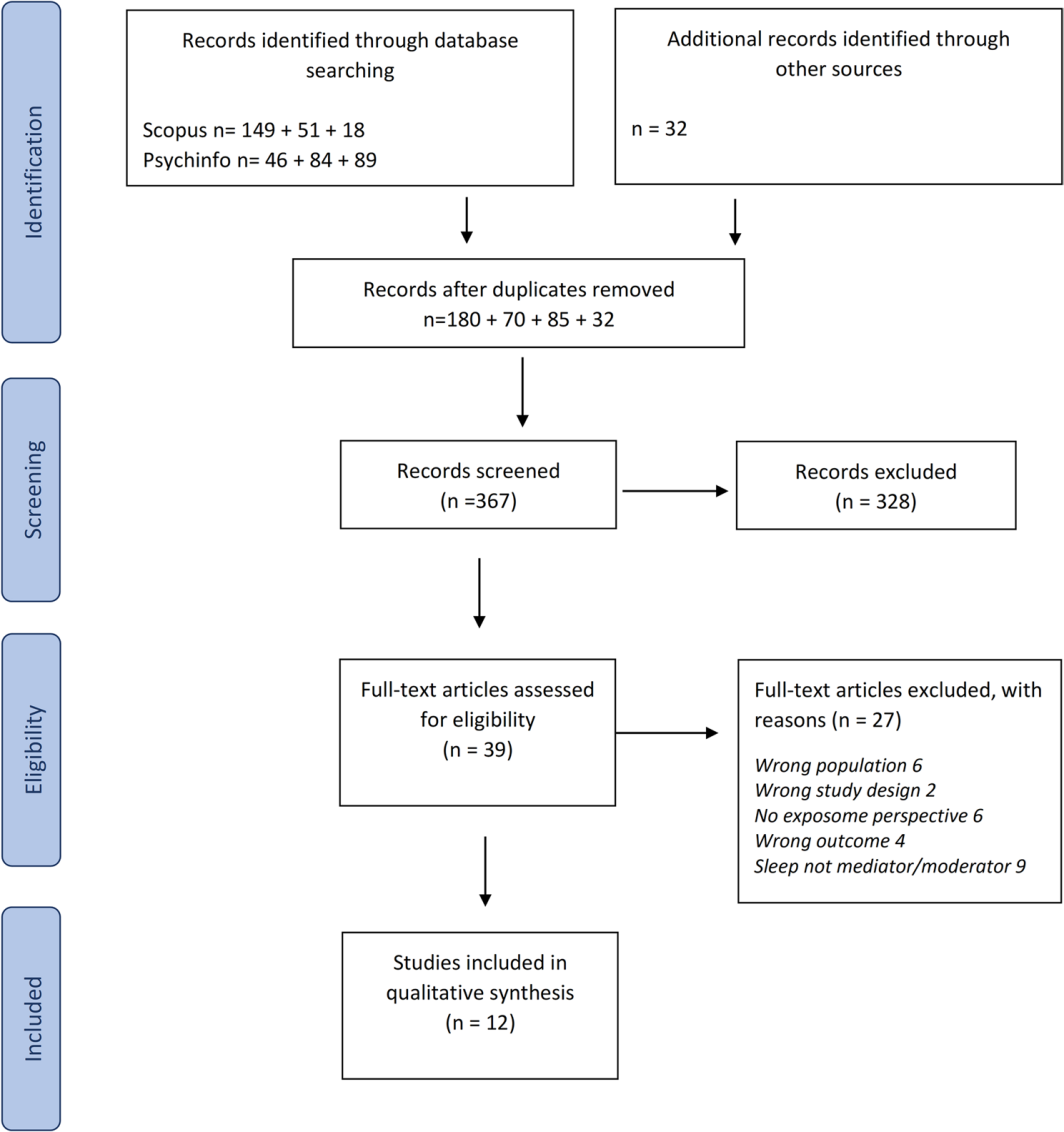
Flowchart of the paper selection process for the scoping review

Figure 1 presents a study selection - flowchart. Searches yielded 367 records after removing duplicates. Following title and abstract screening, we excluded 328 records on relevance. Of the 39 remaining full texts we assessed for eligibility, 27 were excluded. That left us with 12 records of distinct studies (i.e., datasets) [59–70] (see Table S2 for a summary of the studies included in the scoping review). Adjustment strategies varied considerably across studies, with some articulating variables such as co-exposures or modifiers while others were unclear regarding the role of covariates. Most studies included at least some sociodemographic or socioeconomic factors, while fewer explicitly accounted for broader contextual factors such as neighbourhood deprivation and family environment. We therefore interpret findings from individual studies cautiously, particularly where plausibly residual confounding may explain unexpected associations.

### Study design, exposures and outcomes

**Fig. 2:**
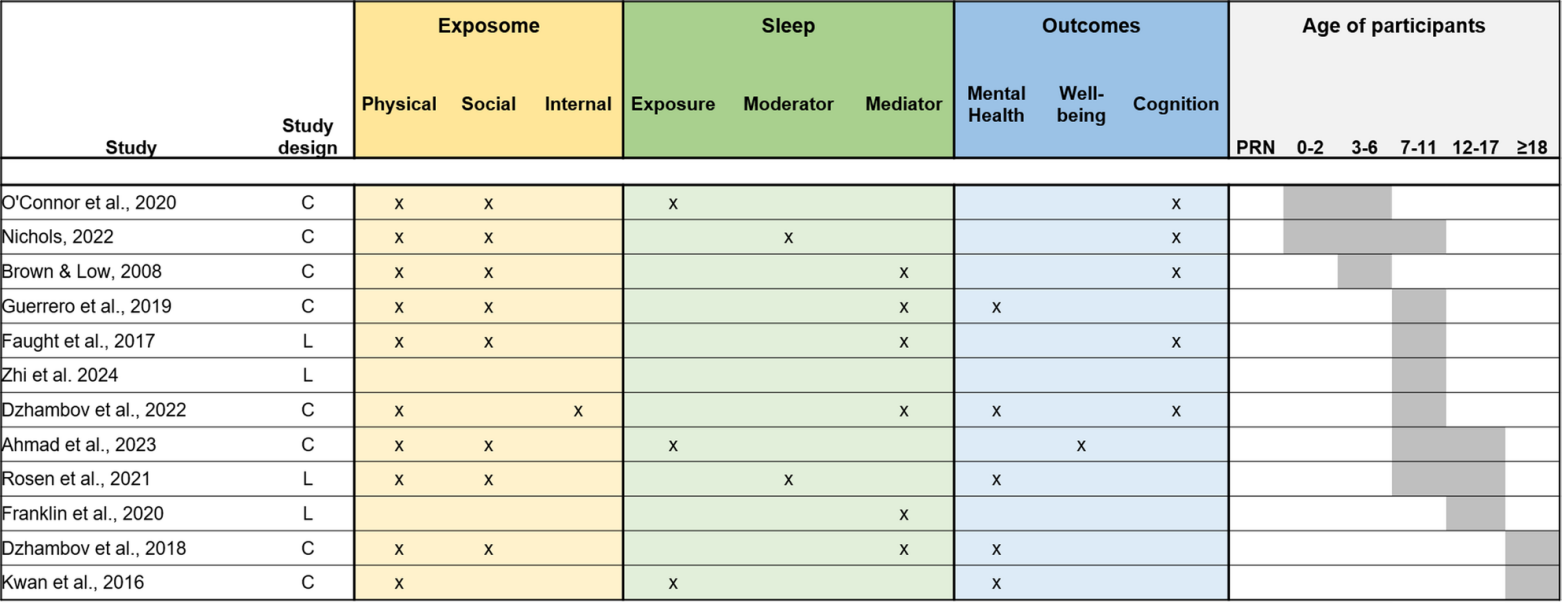
Summary of included papers ordered based on the age groups studied. We highlight the age-groups in focus, the trends regarding outcomes in focus, the use of a life-course perspective and the approach used in relation to multiple exposures and to sleep. Study design: C cross-sectional; L longitudinal

All studies were from high-income regions or countries (6 USA, 2 Canada, 3 Europe, 1 Australia). Studies included children and adolescent samples from 2 to 21 years of age (see Fig. 2). However, most studies included children aged 7–11 years. Only one study analysed the importance of perinatal exposures [65] while no studies explicitly included the prenatal period alone. Included studies were mostly cross-sectional [59, 60, 62–64, 68–70], with only four longitudinal studies that had follow up periods ranging from 6 months to 2 years [61, 65–67]. Most studies used a life-course perspective in their rationale and discussion, considering the long-term effects of exposure on future outcomes as well as the long-term consequences of previous outcomes on future well-being, cognition, and health. Yet evidence on critical windows or on cumulative effects over the life-course was not reported.

Except for Zhi et al. [65], who examined both mental health and cognitive ability longitudinally, studies tended to focus on either mental health or cognition. Cognition was more commonly assessed in early and middle childhood (≤ 10–11 years) [60, 61, 64], whereas studies of children aged ≥ 7 years primarily examined mental health diagnoses or symptoms [63, 70]. Five studies investigated mental health outcomes: general mental health or psychological distress [63, 70], behaviours and unhealthy behaviours [62, 69], and internalizing and externalizing psychopathology [66]. Four studies focused on cognitive outcomes, including academic achievement [61], executive function [68], and psychosocial stress related to the built environment [67]. One study examined well-being [59].

Physical exposures investigated were health behaviours (diet, screen time, physical activity) [59, 61–64], indoor environmental quality (e.g., background noise, second-hand smoke) [60], and outdoor environmental quality (noise, air pollution, nature, restorative qualities) [70]. Franklin et al. [67] specifically examined artificial light at night, near-roadway air pollution, and green space. Social exposures were predominantly sociodemographic and socioeconomic factors, typically treated as confounders and once as an effect modifier [59]. Other social exposures included chaotic living conditions [60] and neighbourhood social cohesion [70]. Zhi et al. [65] uniquely integrated an internal exposure, functional network connectivity derived from resting-state fMRI, also including environmental and social exposures to predict cognitive and mental health outcomes. Rosen et al. [66] uniquely examined pandemic-related stressors.

All studies adopted a multi-exposure approach, although discussions frequently considered exposures individually (Table 3). Interactions were explored using latent class analysis, cumulative indices, structural equation modelling, and multivariate regression. Sleep was assessed exclusively via parent- or child-reported questionnaires, with sleep duration being the most common measure. Three studies conducted formal mediation analyses using parent-reported poor sleep of their children [60], adolescent-reported poor sleep [70], or parent-reported sleep duration [62], all supporting a mediating role of sleep. Only one study assessed both sleep duration and poor sleep [59], and none used objective sleep measures such as actigraphy or polysomnography.

**Table 3:** Summary of exposome, sleep and outcome measures of studies included in the synthesis

	Exposome				Sleep		Outcomes		
Study	Physical	Social	Internal	Multiple exposure analysis	Sleep	Modelled as:	Mental health	Well-being	Cognition
[64] *N* = 1650 4 yrs old	Diet, screen time, physical activity			Multi-variable regression	Sleep duration	Sleep as exposure			Neuro-psychologic develop-ment
	*Food index questionnaire*,* survey questions for physical activity and screen use (how many hours per day on TV or videos): created a childhood healthy lifestyle score*				*Interview: “how long the child sleep daily at night and during naps” (parent reported)*		*Mccarthy Scales of Children’s Abilities (MSCA)*		
[68] *N* = 1180 2 and 8 yrs old	Background TV exposure during various activities	Racial/ethnic background, number of children in the household, maternal age, maternal education, single parent status, socioeconomic status		Multi-variable regression	Sleep during background TV	Moderator			Executive function
	*24-h activity diary*,* parents’ survey*,* cumulative risk index*				*24-h activity diary*		*Behaviour Assessment System for Children (BASC2).*		
[60] *N* = 96 8–10 yrs old	Residential crowding, noise	Family instability		Multi-variable regression	Poor sleep	Mediator – Sobel’s test for mediation			Helpless ad hopeless in relation to academic challenge, Child verbal abilities
	*Demographic interview for caregivers*				*Sleep habits questionnaire (SHQ): Problems related to routines*,* timing*,* nighttime waking*,* daytime sleepiness (caregiver reported)*		*Puzzle and tower tasks*,* peabody picture vocabulary test-III*		
[62] *N* = 11,875 9–10 yrs old	Types of screen use, e.g., television, video games, social media	Content of screen use (mature-rated video games, R-rated movies).			Sleep duration	Mediator	Problem behaviours		
	*Survey*,* habitual physical activity*,* Harvard food frequency questionnaire for youth/adolescents*				*Parent Sleep Disturbance Scale for Children*		*Child Behaviour Checklist (CBCL)*,		
[61] *N* = 4253, 10–11 yrs at baseline	Diet, screen time – tv, physical activity			Mixed effects multi-variable logistic regression	Sleep duration	Sleep as exposure			Academic achievement
	*Survey*,* habitual physical activity*,* Harvard food frequency questionnaire for youth/adolescents*,* parents’ survey about screen use - watching TV*				*Parents’ survey about wake-up and bedtimes for weekdays and weekends*		*Standardized exams in mathematics*,* reading*,* and writing.*		
[65], *n* = 7655 9–10 yrs at baseline	41 environmental exposures across five domains (perinatal, family, school, neighbourhood, individual lifestyle).		Brain functional network connectivity	Multi-variable regression analysis, mediation analysis	Poor sleep	Sleep as exposure	23 behaviours related to cognitive ability and mental health		23 behaviours related to cognitive ability and mental health
	*Developmental history questionnaire*,* parent demographic survey*,* Youth survey*,* Residential history (geocoded residence: crime report*,* area deprivation index*,* modelled PM2.5*,* estimated lead)*,* MRI*				*Parent sleep disturbance scale for children (26 items)*		*Parent reported Child Behaviour Checklist (CBCL)*,* NIH Toolbox*		
[69] *N* = 1468 6–12 yrs old	Nature exposure (home gardens, distance to nature from home and school), Second-hand smoke		Second-hand smoking, Inflammation	SEM	Poor sleep	Mediator	Behaviour problems		
	*Questionnaires*,* Geocoded residential addresses*,* Maternal reported smoking in the house*,* Biomarkers Children’s urine cotinine and neopterin*				*Falling asleep*,* uneasy sleep and feeling tired in the morning*,* (child reported)*		*Needleman questionnaire (assessed by teachers)*		
[59] *N* = 3127 11–17 yrs old	Diet, physical activity, alcohol, smoke	Gender		LCA cluster followed by regression models	Sleep quality, sleep duration	Sleep as part of exposure		Health related quality of life	
	*Survey*				*Survey*,* “On average how much sleep do you? Get per night?” “During the las month*,* how well do you feel you have slept in general?”*		*Survey*,* Paediatric Quality of Life (pedsql) inventory*,* Subscales: physical functioning*,* emotional functioning*,* school function*,* social functioning*,* and psychosocial health summary*		
[66] *N* = 3545 11–18 yrs old	Pandemic-related stressors (health, social, financial, school, physical environment) and potential protective factors (physical activity, time in nature, time outdoors, screen time, news consumption, sleep quantity, family routines, coping strategies, helping others).	Sickness, death, exposure to COVID-19, Difficult relationship, discrimination, Academic stress, Loss of employment, Food insecurity, financial loss, Income to needs ratio, social capital, Family, and community support		Multi-variable regression, moderation analysis	Sleep quantity	Moderator	Internalize and externalize psychopathology		
	*Parents and youth electronic surveys*,* Cumulative index - COVID-19 pandemic stressors and protective factors*				*CDC recommender guidelines for sleep duration (both parental and child reported)*		*Youth self-report and child behaviours checklist (CBL)*,* SDQ*		
[67] *N* = 2290, 13-14yrs baseline	Artificial light at night (ALAN), near-roadway air pollution (NRP), noise, green space, second-hand smoke			Multi-variable mixed-effects models, modification, and mediation analyses	Sleep duration	Mediator	Childhood psychosocial stress measured by the		
	*Child’s Survey supervised*,* Geocoded residential address: Modelled air pollution*,* Modelled traffic noise*,* ALAN 750-m satellite*,* NDVI (world atlas; satellite)*				*Sleep duration*,* trouble going to sleep*,* trouble staying asleep.*		*Perceived Stress Scale (PSS-4).*		
[70] *N* = 720 12–18 yrs old	Residential noise and air pollution, Neighbourhood restorative quality, Physical activity, Environmental annoyance	Social cohesion, sociodemographic		SEM	Poor sleep	Mediator	General health		
	*Geocoded residential addresses*,* Land use regression model for noise (laeq) and NO2*,* Perceived restorativeness scale (PRS)*,* Short questionnaire to assess health-enhancing physical activity*,* Self-reported Noise*,* vibration*,* air pollution annoyance*,* Perceived neighbourhood social cohesion questionnaire.*,* Perceived individual-level economic status*				*Self-reported sleep disturbance*,* “How often do you have trouble falling asleep at night?” “How often to you wake up in the middle of the night?”*		*GHQ*,* symptoms in the past 12 months*		
[63] *N* = 912 14–18 yrs old	Health-risk behaviours (insufficient physical activity, poor diet, insufficient sleep, smoking, marijuana use, illicit drug use, binge drinking, risky sexual behaviours)			LCA	Insufficient sleep	Sleep as exposure	Mental health		
	*Survey*				*Self-reported: “On how many of the past 7 days did you get enough sleep*,* so you felt rested when you woke up in the morning?”*		*Survey: within the last 12 months “have you been diagnosed or treated by a professional for depression or anxiety? Have you ever felt things were hopeless*,* felt exhausted*,* felt very lonely*,* felt incredibly sad felt overwhelming anxiety?”*		

#### Early childhood

For the youngest age groups studied, 2–6 years, chaotic living conditions predicted helpless/hopeless responses to cognitive tasks, and importantly poor sleep, including extensive questions on insomnia-relevant variables, partially mediated this relationship [60]. Another larger study, among 4 year-olds, showed that the total index of health behaviours (Diet, Screen time, Physical activity) and sociodemographic factors (Parental education, Social class) were not related to McCarthy Scales of Children’s Abilities (MSCA) [64]. MSCA is an instrument measuring cognitive ability in six domains: verbal, perceptual performance, quantitative, general cognitive, memory and motor In the same study, a higher physical activity and less than 1 h screen time was unexpectedly associated with lower MSCA scores, indicating poorer cognitive ability, while diet and sleep time > 10 h per day were not associated with poor cognition. DL Nichols [68] investigated the effect of background TV during sleep, predicting poorer executive function among pre-school and school-age children (2 to 8 years old). Cumulative risks (e.g. income to needs, mothers age at birth, more than three siblings, hours of background TV), moderated the association between background TV (BTV) and executive function. The directions and effects were related to age, activity during background TV, and high and low risk children. Children of high socioeconomic risk were exposed longer time for BTV while asleep. In addition, BTV during socialising or doing schoolwork was associated with poorer executive functions for socio-economic low risk school-age children, while the reverse (better executive functions) was found for school-age children of high socio-economic risk families suggesting differential susceptibility. Finally, for preschoolers greater BTV exposure while playing alone predicted poorer executive functioning regardless of socio-economic conditions Importantly, BTV exposure may also be a marker ofhousehold circumstances, including routines, crowding, caregiving demands, parental work patterns, or other unmeasured socioeconomic and family-contextual factors. Although the study accounted for several sociodemographic risks, residual confounding cannot be excluded.

#### Early to middle childhood

For the age groups 7 to 11 years, one study found that sleep duration partially mediated the association between screen time and behavioural problems as measured by Child Behaviour Check List (CBCL) [62]. Longer screen time and shorter sleep duration was associated with greater severity of behavioural problems although the effect sizes were small. Another study within the same age group found a significant relationship between a cumulative index of multiple lifestyle behaviours including sleep and academic outcomes [61]. The higher the number of recommended lifestyle behaviours the higher the likelihood of academic achievements, in maths, reading and writing. In addition, meeting the recommendations for screen time and sleep was associated with improved academic achievement. Zhi et al. [65] examined longitudinally how brain functional network connectivity (FNC) and 41 social and physical exposures affected behaviours related to cognitive ability and mental health. Healthy perinatal social and physical environments were highly important for increased cognitive ability, whereas specifically poor sleep, family conflicts, and adverse school environments increased the risk of poor mental health. They further found that FNC alone predicted cognitive abilities, while FNC along with environmental exposures predicted mental health. Complementing these findings, Dzhambov et al. [69] in a cross-sectional study among 8–12-year-old schoolchildren found that school proximity to nature and the presence of home gardens were associated with fewer behavioural problems, whereas proximity to residential nature beyond the garden was unexpectedly associated with more behavioural problems. Poor sleep did not mediate the association between nature exposure and behavioural outcomes. The unexpected association for residential nature beyond the garden may reflect residual confounding or contextual differences between neighbourhoods, such as urbanicity, land use, deprivation, traffic-related exposures, perceived safety, accessibility, or the quality and usability of nearby green space.

#### Adolescence and early adulthood

Several studies present consistent evidence highlighting the importance of sleep in adolescent health, especially regarding the influence of environmental and psychosocial stressors [59, 66, 67]. Ahmad et al. [59], analysing data from 14–15-year-olds in the Longitudinal Study of Australian Children, identified distinct lifestyle behaviour clusters using latent class analysis. Poor sleep quality was prominent in clusters associated with higher health risk behaviours and poorer outcomes, including obesity, lower self-rated health, and reduced Health-Related Quality of Life (HRQoL), with females being at higher risk. In a cohort of 13–16-year-olds. Franklin et al. [67] found that environmental exposures such as artificial light at night (ALAN), near-roadway air pollution, and second-hand smoke were associated with increased psychosocial stress. Sleep duration partially mediated the relationship between stress and both ALAN and green space, suggesting that sufficient or insufficient sleep may, respectively, buffer or exacerbate environmental stress effects. Rosen et al. [66] examined the impact of pandemic-related stressors on internalising and externalising symptoms in children and adolescents (7–15 years). Adequate quantity of sleep emerged as a protective factor, with those meeting recommended sleep duration guidelines showing fewer psychopathological symptoms. Protective effects were also observed for structured routines, time in nature, and lower screen and lower news/media exposure of the covid pandemic.

Finally, for adolescents more than or equal to 18 years old, or early adulthood, one study [70] found that residential noise level (traffic, neighbourhood, and industrial) was related to mental ill health, both directly and indirectly via annoyance to environmental factors, neighbourhood restorative quality, and physical activity. Sleep was not associated with noise exposure but was associated with mental health via environmental annoyance. In the same study, air pollution was associated with mental health indirectly via annoyance and lower restorative quality, and with lower physical activity. Environmental annoyance and neighbourhood restorative quality and physical activity were thus key mediators. MY Kwan, KP Arbour-Nicitopoulos, E Duku and G Faulkner [63] classified a student sample based on their health behaviours into three groups: high risk, typical and low risk. The high-risk students representing around 20% of the study population reported significantly higher levels of stress than the typical students. Low-risk students reported less stress, fatigue, and psychological distress in relation to the high-risk students. No mediation or moderation analyses was performed on the specific influence of sleep. Noteworthy, the sample did only include around 28% of the target study population due to low response rate.

**Fig. 3:**
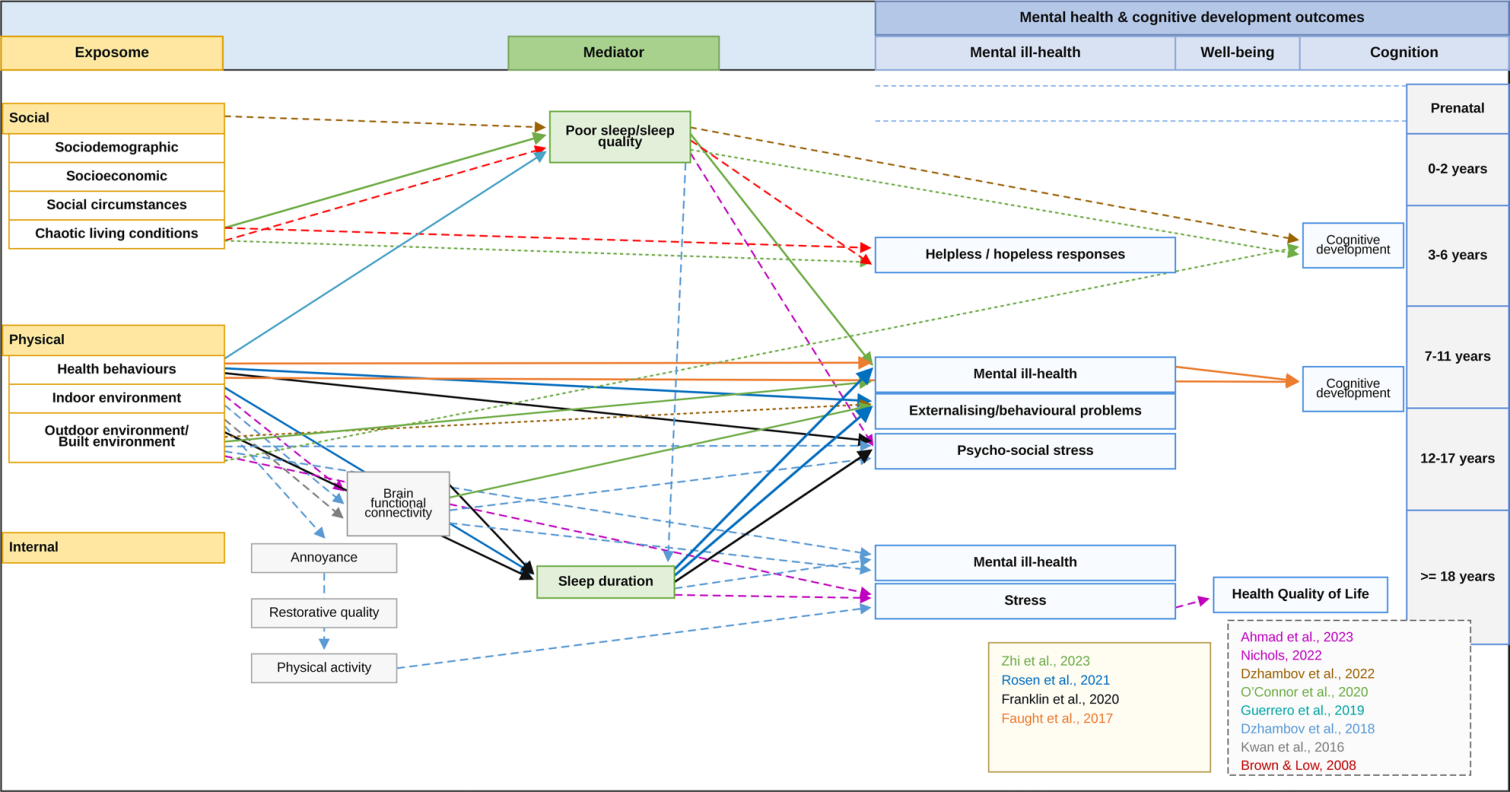
Pathways illustrating the interlinkages of the exposome, sleep and mental health and cognitive developmental outcomes. Solid lines depict findings from longitudinal studies, and dashed lines findings from cross sectional studies. Mediation analyses performed within cross sectional studies are denoted by * after references in the box with references

Figure 3 illustrates the interlinkages of the exposome, sleep and mental health and cognitive outcomes. Three of the four longitudinal studies examining a mediating effect confirmed such pathway. These three studies included a diverse range of exposures ranging from supportive exposures such as green space and time outdoors (Rosen et al. [66]), to adverse exposures in the five domains: perinatal, family, neighbourhood, school, and lifestyle in the comprehensive study by Zhi et al. [65]. Pandemic-related stressors related to health, relations, financial insecurity, crowding, noisy home conditions for schoolwork, screen usage were in addition explored by Rosen et al. [66], and environmental pollutants by Franklin et al. [67]. Furthermore, three cross-sectional studies supported a potential mediating role of sleep in the linkage of exposome and mental health/cognition, with Brown and Low [60] linking chaotic living condition to helpless responses to academic challenges, health behaviours, in particular screen time and content type, being linked to behavioural problems (Guerrero et al. [62]) and residential noise annoyance and air pollution being associated with mental health (Dzhambov et al. [70].

### Additional evidence from studies exploring partial pathways

**Fig. 4:**
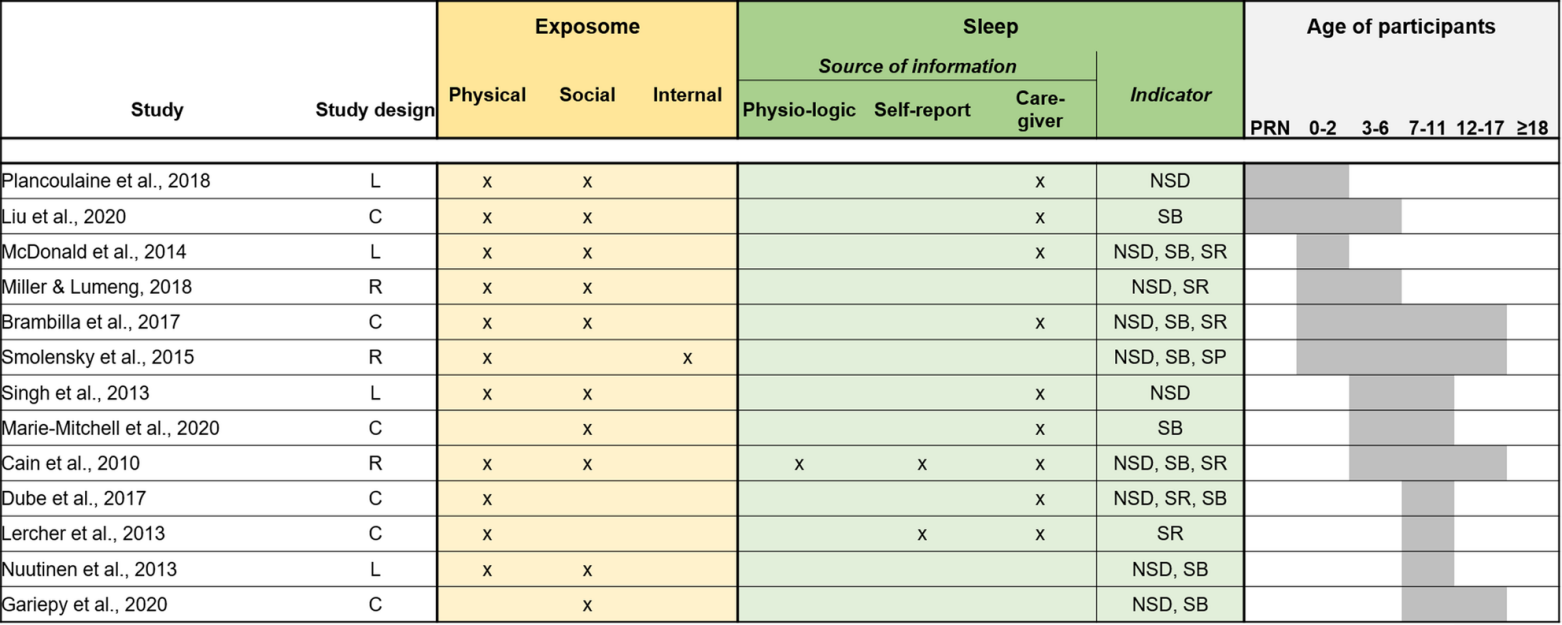
Summary of the studies revised for the partial evidence from exposome to sleep. L=Longitudinal, C=Cross-sectional, R=Review. NSD= Nighttime sleep duration, SB=sleep behaviour, SR= sleep restoration, SP=poor sleep

**Fig. 5:**
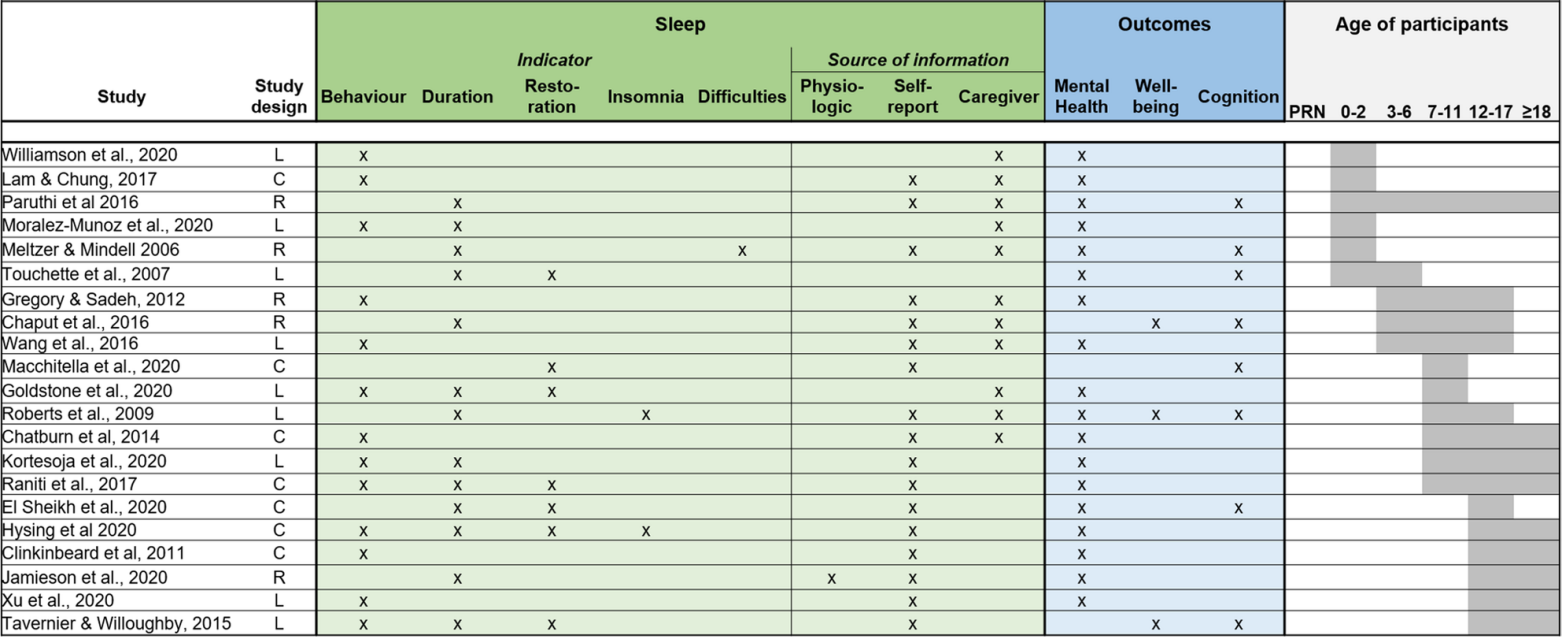
Summary of the studies revised for the partial evidence from sleep to mental health. L=Longitudinal, C=Cross-sectional, R=Review

To deepen the understanding of the pathways linking the exposome to sleep and sleep to mental health and cognitive development, studies including these partial links were reviewed. In total 34 studies were reviewed with 13 studies on the exposome–sleep relationship (3 reviews, 6 cross-sectional studies, and 4 longitudinal studies; see Fig. 4 and Table S3 for a summary) and 21 studies on sleep and mental health/cognitive outcomes (5 reviews, 7 cross-sectional studies, and 9 longitudinal studies; see Fig. 5 and Table S4 for a summary).

#### Exposome and sleep

##### Early childhood

Several studies examined how early-life exposures (prenatal to 2 years) influenced sleep later in childhood. Sociodemographic characteristics of the mother, such as older age, lower educational attainment, and minority ethnic background, were associated with shorter sleep duration in children Child-specific factors, including low birth weight, male sex, and being a firstborn, were also linked to reduced sleep duration [71–73]. Additionally, maternal depressive symptoms during the pre- and postnatal periods were associated with increased poor sleep in preschool-aged children [18].

##### Early to middle childhood

In early to middle childhood (3–11 years), both social and physical environmental exposures were found to influence sleep. Adverse childhood experiences, such as parental divorce, abuse, or having a mentally ill family member, were predictive of poorer sleep behaviour in children aged 5–11 years [74]. Indoor environmental factors also played a significant role. For example, children living in homes with high levels of noise and chaos, or lacking adequate sleep spaces, experienced poorer sleep health [75]. Exposure to second-hand smoke was associated with insufficient sleep among children aged 6–11 years [76]. Additionally, self-reported traffic noise exposure was linked to poorer sleep behaviour in children aged 8–11 [77].

##### Adolescence

Among adolescents (12–17 years), physical exposures, particularly electronic media use, were the most frequently studied. Screen time during the day and before bedtime, as well as having access to electronic devices in the bedroom, were consistently associated with shorter sleep duration, delayed sleep onset, increased sleep disturbances, and poorer sleep quality [72, 73, 76, 78–81]. These effects were attributed to sleep displacement, increased arousal, and circadian rhythm disruption due to blue light exposure [78].

In addition to screen use, physical inactivity was also linked to shorter sleep duration in adolescents aged 6–17 years. Neighbourhood characteristics, such as limited access to amenities like parks and libraries, were associated with shorter nighttime sleep [76]. Once again, physical activity and amenity access may implicitly capture a wider range of contextual factors, including neighbourhood socioeconomic conditions, perceived safety, walkability, social cohesion, family resources, and opportunities for structured leisure activities. Sleep duration varied widely across European and North American countries but not clearly according to Socio Economic Status (SES) and gender. Older adolescents have consistently shorter sleep duration than younger adolescents [82].

#### Sleep and mental health/cognitive development

##### Early childhood

Six studies were identified for early childhood (0–2 years), including two longitudinal studies, two cross-sectional studies, and two reviews. AA Williamson, JA Mindell, H Hiscock and J Quach [83] conducted a longitudinal study in which parents rated whether their child’s sleep was problematic. Children were followed annually from infancy to age 10–11. Based on sleep trajectories, five groups were identified. Children with persistent sleep problems exhibited the most significant impairments across mental health, well-being and cognitive outcomes except for perceptual reasoning, and lower academic achievement scores. Poor sleep that emerged after age 4–5 years was associated with increased internalising and externalising symptoms and reduced quality of life, and small yet significant reduced academic scores.

Morales-Muñoz et al. [84] assessed sleep behaviour and nighttime sleep duration at multiple time points from 6 months to 5.8 years. Frequent night awakenings at 18 months and irregular sleep at several ages were linked to psychotic experiences in adolescence. Shorter sleep duration and later bedtimes at 3.5 years were associated with borderline personality disorder symptoms. Depression at age 10 mediated the relationship between early sleep disturbances and psychosis at ages 12–13. Further associations between shorter sleep duration and behavioural or mental health problems were found in cross sectional studies. Lam and Chung [85] reported that SES moderated the relationship between sleep behaviour and externalising problems, with stronger effects observed in lower SES groups.

Paruthi et al. [21] highlighted the importance of nighttime sleep for communication, language, and problem-solving development. Shorter sleep in infancy was associated with lower quality of life at age 6–7 and more emotional and behavioural problems at age 5. Meltzer and Mindell [86] similarly emphasized that inadequate or disrupted sleep in childhood is associated with emotional and behavioural difficulties, impaired daytime functioning, and poorer school-related outcomes.

##### Early and middle childhood

One longitudinal study and two reviews were identified for early childhood (3–6 years). Wang et al. [87] followed 2,868 children, with 1,993 completing at least three follow-ups. Sleep was assessed at ages 5, 8, 10, and 14, and mental health at ages 5 and 17. Most children experienced less poor sleep over time. However, about 10% had persistent or increasing sleep issues, which were linked to attention problems, aggressive behaviour, and anxiety or depression (especially in girls) at age 5, and continued behavioural issues at age 17.

Reviews emphasized the need for higher-quality longitudinal research. Chaput et al. [88] found that short nighttime sleep was linked to poorer emotional regulation in about half of the studies, impaired growth in two studies, and increased screen time in all five studies reviewed. However, evidence for associations with cognitive function or quality of life was inconclusive.

The reviews by Chaput et al. [88], Gregory and Sadeh [89] and Paruthi et al. [21] noted that most studies were cross-sectional and relied on self-reports. Longer sleep duration was generally associated with better emotional regulation and academic achievement, though findings on cognition were mixed. One study found that preschoolers who slept less than 11 h had poorer vocabulary. Daytime naps were also important for emotional and cognitive functioning. Another study found that toddlers and preschoolers with less than 10 h of sleep had higher rates of hyperactivity and lower cognitive performance [90].

For school age to middle childhood (7–12 years), Goldstone et al. [91] followed nearly 5,000 children aged 9–10 for one year. Parental reports of disturbed sleep behaviour predicted internalizing, externalizing, and depressive symptoms one year later. Girls were particularly vulnerable to depression following sleep disturbances. Sleep behaviour was a stronger predictor than sleep duration. Cross-sectional findings showed that daytime sleepiness in children aged 7.8–11.2 years was associated with poorer performance in verbal and mathematical tasks, but not in visual-spatial abilities [92]. In a smaller study of 62 children and adolescents aged 7–18, Chatburn et al, [93] found that poor sleep were strongly correlated with lower resilience, which in turn mediated the relationship between sleep issues and internalizing and externalizing problems, including depression and anxiety. Paruthi et al. [21] reported that short sleep duration in children aged 6–13 was associated with emotional dysregulation, irritability, poor peer and family relationships, and lower perceived health.

##### Adolescence and young adulthood

In a large prospective study, RE Roberts, CR Roberts and HT Duong [34] found that adolescents sleeping less than six hours had increased risks of depression and academic difficulties. Short sleep on both weekdays and weekends was associated with poor life satisfaction and mental health. When adjusting for insomnia at baseline, short sleep duration remained significantly associated with academic problems and low grades. Kortesoja, et al [94] identified a bidirectional relationship between poor sleep and psychosocial difficulties in a five-year follow-up of over 3,700 Finnish adolescents. Short sleep duration predicted emotional symptoms, conduct problems, and hyperactivity, while these psychosocial difficulties also led to future poor sleep. Increased sleep deprivation during adolescence (14-18yrs) is linked to higher rates of mental disorders. This relationship may be mediated by delayed brain maturation—specifically, underdevelopment of the uncinate fasciculus—reducing emotional regulation through impaired top-down control [28].

A review by Paruthi et al. [21], focusing on individuals aged 10–19 years, reported that reducing sleep duration by one hour was associated with lower academic achievement, with older adolescents being more affected. The review also identified a U-shaped relationship, where both insufficient and excessively long sleep durations were linked to poorer performance. Additionally, insufficient sleep was associated with heightened risks of depression, self-harm, suicidal ideation, and suicide attempts.

Several longitudinal and cross-sectional studies in late adolescence and early adulthood (15–25 years) explored the complex interplay between sleep and mental health. R Tavernier and T Willoughby [95] conducted a longitudinal study involving 942 university students aged 17–25, revealing a bidirectional association between poor sleep and social ties. Emotion regulation emerged as a key mediator: better sleep—characterized by longer duration, healthier sleep behaviour, and greater restoration—was linked to improved emotion regulation and stronger social connections. In turn, positive social ties enhanced emotion regulation, which subsequently reduced poor sleep.

Xu et al. [96] followed adolescents aged 15–24 over four waves at three-month intervals to examine the mediating roles of emotional exhaustion and sleep-related worry in the relationship between sleep behaviour and mental health. Their findings showed that sleep disturbance indirectly influenced depressive symptoms through the mediators - emotional exhaustion and sleep-related worry- while the direct effect of sleep disturbance on depression was not statistically significant.

Raniti et al. [30] reported that short sleep duration and poor sleep quality were associated with increased depressive symptoms. Furthermore, their mediation analysis revealed that sleep quality—assessed via self-report—significantly mediated the relationship between age and depressive symptoms. Other sleep parameters, such as sleep disturbance, sleep efficiency, and sleep onset latency, did not show significant mediation. Conversely, depressive symptoms significantly mediated the relationship between age and various sleep outcomes, reinforcing the bidirectional nature of sleep and mental health during this developmental stage.

Additional cross-sectional studies support the interlinkages of mental disorders and poor sleep. El-Sheikh et al. [97] linked short sleep duration to internalizing and externalizing symptoms as well as lower cognitive performance, with stronger effects observed in lower socioeconomic groups. Hysing et al. [98] confirmed a higher prevalence of insomnia and short sleep duration among all groups of psychiatric disorders although highest among adolescents with depression, while Clinkinbeard et al. [99] highlighted a specific association between short sleep duration and delinquent behaviour.

## Discussion

Several noteworthy findings emerged from the review. The hypothesis that poor sleep and/or sleep duration may mediate the association of external exposome and mental health and cognitive development in children and adolescents was largely substantiated. While it is previously well shown that a substantial proportion of depressed adolescents commonly have poor sleep bidirectionality cannot always be excluded [100]. However, emerging longitudinally derived evidence suggests that sleep disturbance more often precedes the onset of depressive symptoms rather than resulting from them. For example, short sleep duration was identified as a prospective predictor of depressive symptoms in adolescents [101] and other longitudinal studies point to self-reported sleep disturbance predicting the onset of depressive disorders [33, 102]. However, where psychological issues are assessed as later diagnoses, latent or subclinical problems may already have been present at the time of sleep assessment. The interpretation that poor sleep precedes psychological issues is therefore more compelling when psychological symptoms or disorders were assessed at baseline and taken into account. Furthermore, a review of 23 studies among individuals aged 12 to 20 years provided further support from longitudinal and treatment studies that sleep disturbance tended to proceed the development of depression, with limited support for the reverse temporal association [31]. Given the potential health consequences of poor sleep in these age groups there is an urgent need for additional longitudinal studies, to inform preventive interventions addressing their residential environments.

Among studies assessing the full pathway, the potential mediating effect of sleep was primarily demonstrated for mental ill health, whereas evidence for cognitive development was sparse. The latter was unexpected given the comparatively large number of previous studies highlighting the crucial role of sleep quality and quantity for neurodevelopmental growth and functions vital for learning, alertness and concentration [28, 103]. However, additional insight was provided from studies examining partial pathways. Longitudinal studies indicates that poor or short sleep duration in early childhood, during the early school years, and particularly in adolescence and young adulthood, is associated with later cognitive impairments and poorer academic performance. A review [21], focusing on individuals aged 10–19 years, further demonstrated that that both short and long sleep duration was associated with poorer academic achievement, with older adolescents being more affected. These findings underscore the need to additionally study how children’s and adolescents’ social, physical, and internal exposures – their exposome – affects their cognitive development and academic performance, and whether critical periods or age-specific susceptibility can be disentangled.

Additional evidence on partial pathways reinforced the links between exposome, sleep, and adverse mental health and cognitive outcomes. For the partial pathway of sleep and mental health and cognitive outcomes, substantial evidence was provided by large longitudinal studies, showing that children with early sleep disturbances, particularly during their first 10 years of life, had an increased risk of later mental health problems [84]; as well as cognitive and attention difficulties [83, 87]. While the proportion of children with poor sleep tended to diminish with age, a general finding was that a smaller group (around 8–10%) reported persistently higher poor sleep from childhood through adolescence. The observation that sleep patterns acquired at early childhood persist through adolescence for both good and poor sleepers (e.g., Al Mamun et al. [104]), is worth paying attention to from an exposome perspective. A longitudinal study with a shorter follow-up period [91] corroborated these findings with baseline sleep disturbance significantly predicting depression and internalizing and externalizing scores at 1-year follow-up. These results are consistent with evidence that poor sleep in early childhood prospectively predicts later emotional and behavioural difficulties [90].

Other large longitudinal studies of longer [94] and shorter follow-up periods [95, 96] highlight the importance of including emotion regulation or emotional exhaustion as possible mediators in the linkage. The study by [94] demonstrated that short sleep duration predicted emotional and behavioural difficulties across adolescence, which in turn resulted prospectively in poor sleep. These findings suggest an age-related development where sleep and emotional and behavioural difficulties are intertwined in shaping adolescents’ health. Similarly [95, 96], reported that sleep disturbance was associated with depression via emotional exhaustion and sleep related worry.

Regarding exposome and sleep, some important findings were that social exposures during the prenatal period and up to 2 years of age were related to shorter sleep duration for the child later on in life [71–73]. Among school-age children or adolescents, access to and use of electronic media was the most frequently examined physical exposure. The findings suggest that electronic media use, specifically daytime and pre-bedtime screen time, as well as having bedroom access to electronic media, predicted shorter sleep duration, delayed sleep phasing, sleep disturbance, and poorer sleep quality in children and adolescents [72, 73, 76, 78–81]. Proposed mechanisms for this relationship were that media use directly displaced sleep, caused increased mental, emotional or physiological arousal, and that blue light exposure delayed the circadian rhythm [78]. Other health related behaviour such as physical inactivity was seen to be linked to shorter sleep duration in 6-17-year-olds [76]. In addition, cross sectional studies found that second-hand smoke exposure, chaotic home environments, low neighbourhood amenity access, and higher reported noise exposure from traffic, though not the actual noise levels, were related to poor sleep restoration. Associations between sleep and neighbourhood amenities or physical activity may therefore reflect not only the direct influence of activity opportunities or facilities, but also wider social and environmental conditions that shape children’s daily routines, safety, stress, and sleep opportunities.

Given the diversity and complexity of exposures, particularly evident in the longitudinal studies exploring the full linkage via sleep, it is unlikely that any single exposure sufficiently accounts for the observed associations. A more plausible explanation is that the cumulative and interacting effects of multiple exposures, assessed through a multiple-exposure framework, underlies the relationships identified. Historically, most studies have focussed on single exposures and specific outcomes, commonly treating social factors as merely confounders, while interactions or combinations of diverse exposures have less commonly been investigated. However, emerging discussions emphasising the importance of the totality of exposures within a life-course perspective, particularly with regards to the early-age exposures and outcomes [13, 105], underscore the limitations of these simplifications and point to an urgent need of additional studies embracing this broader perspective. Encouragingly, an increasing number of exposome studies have recently addressed children’s and adolescents’ health (e.g. [106–109]. although mental health and cognitive development outcomes remain comparatively less explored [110, 111].

Overall, the findings from this review underscore the importance of adopting an exposome perspective emphasizing totality of exposures in a developmental perspective. Early-life exposures as well as trajectories of key mediators such as sleep may play a critical role in shaping mental health and cognitive outcomes in late childhood or adolescence. The identification of key mediators is particularly valuable, as it may inform the development of targeted and potentially more effective preventive interventions.

### Methodological considerations

Several methodological considerations were identified in accordance with PRISMA-ScR guidance. Study selection required interpretive judgment for sources examining multiple exposures without explicitly adopting an exposome framework; eligibility was based on conceptual alignment rather than terminology. Consistent with the review objectives, only studies examining pathways linking the exposome to mental health or cognitive development via sleep were included.

Operationalization of the life-course perspective varied across sources of evidence, with studies focusing on different developmental periods, including early childhood, adolescence, young adulthood, and the transition to adulthood. This heterogeneity limits comparability across studies.

Sleep measurement relied on commonly available epidemiological indicators, namely sleep duration and self-reported sleep quality or sleep-related behaviours (“poor sleep”) [112]. Although supported by prior evidence linking short or poor-quality sleep to adverse emotional and cognitive outcomes [23, 24], reliance on subjective measures introduces potential measurement bias, and no study included objective sleep assessments.

A further limitation concerns residual confounding in some of the studies. Although several studies accounted for sociodemographic or socioeconomic factors, the degree to which family context, neighbourhood characteristics, co-occurring environmental exposures, health behaviours, and pre-existing psychological symptoms were accounted for varied substantially. This is particularly relevant for unexpected findings, such as associations involving background TV exposure, proximity to residential nature beyond the garden, access to neighbourhood amenities, and physical activity. These exposures may function not only as specific environmental or behavioural factors, but also as indicators of broader social, spatial, and family contexts. Consequently, causal interpretations should be made cautiously, and future exposome studies should more systematically account for correlated exposures and contextual confounding.

Methodological approaches to exposure complexity were limited. Few studies adopted explicit multiple-exposure or cumulative risk approaches [113], interactions between exposures were rarely examined, and social exposures, despite frequent inclusion, were most often treated as confounders rather than core components of the exposome.

Lastly, this scoping review was limited to peer-reviewed articles published in English, and relevant evidence from other languages or the grey literature may therefore have been missed. Inclusion of studies not explicitly using an exposome framework required interpretive judgement during screening, introducing potential subjectivity despite predefined criteria and independent review. In line with PRISMA-ScR guidance, no formal assessment of methodological quality or risk of bias was undertaken, and findings should therefore be interpreted as a mapping of available evidence rather than an evaluation of its strength.

## Conclusion

This literature review highlights emerging evidence of sleep being a critical link in the relationship between exposome and mental health, and cognition in children and adolescents. Although the number of available studies remains limited, the finding that a multitude of exposures may influence children’s and adolescents’ mental health and cognitive development through sleep is of considerable significance. This is important not only at the individual level but also from a public health perspective, as the developing child and adolescent may be particularly vulnerable to sleep disturbances during critical periods of neurodevelopment, potentially leading to adverse consequences for cognitive development, mental health, and academic success later in life. Incorporating a child-centred exposome framework and longitudinal methodologies is essential for advancing our understanding of how sleep interacts with complex environmental exposures to foster mental health and cognitive trajectories across childhood and adolescence.

## Supplementary Information

Supplementary Material 1.

## Data Availability

No datasets were generated or analysed during the current study.
