# Supplementary material for: Sleep as a potential link between exposome, mental health and cognitive development in children and adolescents - a scoping review
Source: Arch Public Health. 2026 Jul 17;84:158. doi: 10.1186/s13690-026-02016-9 (PMC13377782; doi:10.1186/s13690-026-02016-9)
Supplement: Supplementary file 1 — Supplementary Material 1. [file 13690_2026_2016_MOESM1_ESM.docx]

# Supplement

Table S1. Search terms and search block used in scoping review

| **Search blocks** | **Search terms** | |
| --- | --- | --- |
| **Population** | neonat* OR zygot* OR embryo* OR fetus OR foetus OR fetal OR newborn* OR child* OR infant OR infancy OR toddler OR puberty OR pubertal OR early life OR teen* OR youth* OR adolescen* OR juvenile* OR young* OR pre-teen* OR preteen* OR pre-adolescen* OR preadolescen* OR pediatric OR student OR pre-school OR school | |
| **AND** | |  |
| **Exposome** | #1 exposome OR “lifecourse epidemiology” OR co-expos* OR omics  #2 expos* OR environment* OR hazards OR risk OR risks  #3 joint OR combi* OR complex OR synerg* OR additive* OR multi OR multiple OR cumulat* OR collect* OR interact* OR compensat* OR lifetime OR life-long OR totality OR several OR interplay OR holistic OR “more than one” OR comprehensive OR stress* OR overall  #4 nois* OR pollut* OR "particulate matter" OR dust OR vibrat* OR electromagn* OR climate OR weather OR temperature OR humid* OR season* OR water OR food OR nutri* OR diet OR green OR blue OR natur* OR outdoor OR restorative OR mould OR mold OR damp* OR "built environment" OR indoor OR housing OR dwelling OR residential OR urban OR rural OR school OR pre-school OR daycare OR kindergarten  (#1 OR (#2 AND #3)) AND #4  For the (#2 AND #3), proximity operators were used in a way that the search terms in #2 should be maximum 4 words apart from the search terms in #3 | |
| **AND** | |  |
| **Outcome** | mental OR psych* OR internali* OR externali* OR “conduct disorder” OR emotion* OR “affective symptoms” OR mood OR temper* OR sadness OR anxiety OR depress* OR neurodevelop* OR behaviour* OR behaviour* OR neuropsychology OR neurobehavior OR neurobehaviour OR hyperactiv* OR autis* OR personality OR obsessive OR “eating disorder” OR hyperactiv* OR “oppositional defiant” OR p-factor OR tics OR self-harm OR cognit* OR reading OR spelling OR math* or arithmetic* OR literacy OR intellect* OR intelligen* OR iq OR language OR speech OR learn* OR memory OR “executive functions” OR “executive function” OR attenti* OR inattenti*OR competence* OR skill* OR “problem solving” OR well-being OR well-being OR “well being” OR “quality of life” OR “peer-relation” OR “approval seeking” OR empath* OR empower* OR agressi* OR anger OR irritation OR helpless* OR hopeless* OR attachment OR insecur* OR secur* OR powerless* OR addiction OR addicted OR delinquen* OR prosocial OR pro-social OR stubborn OR tantrum OR antisocial OR SDQ OR ((school OR academic OR educat*) AND (achievement OR attainment OR perform* OR grades))  For the search ((school OR academic OR educat*) AND (achievement OR attainment OR perform* OR grades)), proximity operators were used in a way that the search terms in the first bracket should be maximum 4 words apart from the search terms in the second bracket. | |
| **AND** | |  |
| **Mediation** | mediat* OR pathway* OR mechanis* OR “structural equation” OR ”path analysis” OR moderat* | |
| **AND** | |  |
| **Sleep** | Sleep* OR “sleep duration” OR insomnia OR parasomnia* OR nightmares OR “night terrors” OR dyssomnia* OR REM OR non-REM OR polysomnogr* OR awakening OR arousal OR “circadian rhythm” OR bedtime resistance OR wake OR wakening* OR nocturnal OR overnight OR evening OR morning OR tiredness OR fatigue OR daytime functioning | |

Note. Underlined search terms were added for the second search on Oct 5, 2020. In the Specific social and internal exposures were considered at a later stage, during screening the retrieved study records.

Table S2. Summary of papers included in the scoping review

| **Study** | **Exposome** | | | | **Outcomes** | **Sleep** | **Analysis** | **Results** |
| --- | --- | --- | --- | --- | --- | --- | --- | --- |
|  | **Physical** | **Social** | **Internal** | |  |  |  |  |
| (Zhi et al., 2023)  Longitudinal  9-10 yrs old at baseline  N=7655  USA (ABCD Study)  Cross-validation UK biobank study N=20852 | 41 environmental exposures across 5 domains:  - Perinatal (e.g., breastfeeding)  - Family (e.g., income, conflicts)  - Neighbourhood (e.g., security, deprivation)  - School (e.g., climate, engagement)  - Lifestyle and events (e.g., screen use) | | | Brain functional connectivity (FNC) derived from MRI | Cognition: 10 abilities (NIH Toolbox)  Mental health: 13 behavioural and psychopathology measures | Poor sleep (parent reported) | Linear mixed-effects models | Healthy perinatal development (e.g. breastfeeding duration, early verbal development) was protective for cognitive ability.  Poor sleep, family conflicts, and adverse school environments increased the risk of poor mental health.  Sleep problems emerged as one of the strongest predictors of mental health, but not cognition.  Environmental exposures had greater predictive power than brain connectivity alone. FNCs mediated the environment-behaviour associations. |
| (Ahmad et al., 2023)  Cross-sectional  14-15 yrs old  Australia | Life-style factors (e.g., physical activity, screen use, gaming, diet, alcohol use, smoking | Sex (male and female) | NA | | Self-rated health  Health-related quality of life | Sleep duration  Sleep quality | Latent class analysis: classes used as predictors in regression models | Clusters characterised by poor sleep quality and/or short sleep duration, low physical activity and high sedentary behaviour, showed higher odds of poor outcomes.  Poor sleep co-occurred with other unhealthy behaviours, rather than acting independently.  Clustering differed between sex: male seems to be more vulnerable. |
| (Dzhambov et al., 2022)  Cross-sectional  8-12 yrs old  Tyrol region Austria, Italy | Outdoor environment (e.g., home gardens, distance to nature)  House type  Second-hand smoke | Socio demographic |  | | Behaviour problems | Sleep quality  Sleep restoration | SEM | Home gardens and proximity to nature at school were associated with fewer behaviour problems.  The presence of a home garden indirectly reduced behaviour problems through lower second-hand smoke exposure.  Sleep did not mediate the relation between home gardens and outcome.  Unexpected associations were found between higher residential proximity to nature and worse behaviour problems. |
| (Nichols, 2022)  Cross sectional  2--8 years old  USA | Indoor environment quality: Background TV (framed as chronic noise exposure) | Cumulative index (e.g., maternal education, income, age) | NA | | Executive functions (EF) | Sleep quality | Linear regression models with cumulative risk as moderator | Children at high socioeconomic risk were exposed to more minutes of background TV (BTV) while sleeping.  For preschoolers, greater BTV exposure while playing alone predicted poorer executive functioning.  For school-age children, in low-risk families, BTV during socialising or academic activities was associated with poorer executive functioning. In high-risk families, these same contexts were associated with relatively better executive functioning, suggesting differential susceptibility. |
| (Rosen et al., 2021b)  Longitudinal  7-15 yrs old  USA | Cumulative index  COVID-19 pandemic stressors and protective factors | | |  | Internalizing, externalizing behaviours  Youth self-report (YRS) | Sleep duration | Linear regression models | Getting recommended amount of sleep during the pandemic was marginally associated with lower levels of externalizing behaviours 6 months later.  Structured daily routines, low passive screen time, and limited news consumption showed stronger and more consistent protective associations than sleep.  The association between pandemic-related stressors and outcome was reduced among youths with low screen time and low news media exposure. |
|  | Indoor environment  Health behaviours (e.g., screen time, physical activity)  Time outdoors and in nature | Difficult relationship  Discrimination  Academic stress  Socioeconomic  Social capital  Family and community support | NA | |  |  |  |  |
| (Franklin et al., 2020)  Longitudinal – 2yrs  13-16 yrs old  USA | Outdoor and indoor environment quality (e.g., ALAN, air pollution, traffic noise, green space, second-hand smoke) | Neighbour-hood socio-economic context |  | | \| Perceived psychosocial stress  (PSS-4) \|  \|  \| \| --- \| --- \| --- \| | Sleep behaviour  Sleep duration | Regression models with interaction and mediation analysis | Higher exposure to ALAN, air pollution, and second-hand smoke were each independently associated with higher perceived stress.  Greater residential green space was associated with lower perceived stress, partially offsetting the effects of urban environmental stressors.  Sleep duration partially mediated the associations between stress and both ALAN and green space  The association between ALAN and stress was stronger in lower-income neighbourhoods. |
| (O’Connor et al., 2020)  Cross-sectional  4 yrs old  Spain | Health behaviours (e.g., screen time, physical activity, diet quality)  Exposure to tobacco smoke | Socio-economic position  Exposure to tobacco smoke | NA | | Cognition | Sleep duration | Linear regression models with a Child Healthy Lifestyle Score (CHLS) | The **combined healthy lifestyle score** (diet, sleep, screen time, physical activity) was **not associated** with general cognitive performance at age 4.  Unexpectedly, higher physical activity and lower screen time were associated with lower cognitive scores.  Associations did not differ meaningfully by sex, cohort or in families of lower social class. |
| (Guerrero et al., 2019)  Cross-sectional  9-10 yrs old  USA (ABCD study) | Health behaviours (e.g., scree time, content)  Somatic vulnerability | Family socio-economic characteristics |  | | Emotional and behavioural problems anxious/depressed, withdrawn/depressed, somatic complaints, social, thought, attention problems, rule-breaking and aggressive behaviour) | Sleep duration (parent reported) | Mediation analysis | Greater time spent in screen time, mature-rated content and lower sleep duration were associated with greater problem behaviours among children.  Sleep duration partially mediated the relationship between screen time (types and content) and behavioural problems, although indirect effects were small relative to the strong direct associations between sleep and behaviour.  Findings support sleep as a key protective factor in the context of screen-related behavioural risk. |
| (Dzhambov et al., 2018)  Cross-sectional  Mean age:21  Range 18-35 yrs old  Bulgaria | Health behaviours  Outdoor environment quality (e.g., traffic noise, air pollution, ne, restorative quality)  Time spent at home/day  Built environment  Urbanicity | Socio-demographic  Socio-economic  Social circumstances  Neighbourhood social cohesion |  | | Mental health (e.g., general health, anxiety/depression symptoms) | Sleep behaviour | Structural equation modelling (SEM) | Increased residential noise was associated with poorer mental health through pathways involving environmental annoyance, lower restorative quality, reduced physical activity, and sleep disturbance.  Air pollution was indirectly associated with mental health through similar pathways. |
| (Faught et al., 2017)  Longitudinal  1 yr. follow up  10-11 yrs old  Canada | Health behaviours (e.g., screen time, physical activity, diet)  Built environment  Urban/rural  Somatic vulnerability | Socio-demographic  Socio-economic |  | | Academic achievement | Sleep Duration (parent reported)  Sleep behaviour | Mixed effects logistic regression models with students clustered in schools | Health behaviours, not body weight status, were strongly associated with academic performance.  Meeting multiple lifestyle behaviour recommendations had a greater impact on academic achievement than individual behaviours.  Multiple healthy lifestyle behaviours together, rather than any single behaviour or body weight, are most strongly associated with academic success. |
| (Kwan et al., 2016)  Cross-sectional  Mean age = 20.9 SD 3.7 years old  Canada | Health behaviours (e.g., physical activity, diet) | Substance use  Risky sexual behavious | NA | | Anxiety  Depression  Stress  Fatigue  Psychological distress | Sleep duration | Latent class analysis | Three distinct behavioural profiles with varying from typical to high-risk.  The higher risk group (multiple health risks including sleep) more stress but not significant more fatigue, anxiety, depression or distress.  Vast majority of students, regardless of risk group had low probabilities of engaging in health promoting behaviours. |
| (Brown and Low, 2008)  Cross sectional  3-5 years old  N=96  USA | Indoor environment quality (e.g., noise, crowding)  TV noise (>4 hr) day when child is home. | Socio-demographic  Socio-economic  Social circumstances (e.g., family instability) |  | | Child verbal ability  Helpless/hopeless responses to academic challenge | Sleep behaviour  Poor sleep  Sleep restoration | Linear Regression models  Mediation analyses (Sobel test) | Chaotic living conditions predicted helpless/hopeless responses to academic challenge, with poor sleep partially mediating this relationship.  Results supported additive and mediation models between chaotic living conditions and poor sleep rather than moderation. |

Table S3. Summary of the studies on partial links exposome to sleep

| **Study** | **Design** | **Key Exposures** | **Sleep Outcomes** | **Age Range** | **Key Findings** |
| --- | --- | --- | --- | --- | --- |
| (Plancoulaine et al., 2018) | Longitudinal | Parental education, household income, Maternal characteristics, child sex, temperament, dietary patterns, screen time (TV) | Nighttime sleep duration, sleep trajectories | 2-6 yrs | Identified 5 sleep duration trajectories. Short or worsening sleep trajectories were most strongly associated with modifiable behaviours and routines (night-waking, parental sleep-onset practices, TV time, diet patterns, night feeding), plus some family/maternal factors (first-born boys, maternal age, maternal work, smoking/depressive symptoms). |
| (Liu et al., 2020) | Cross-sectional | Maternal mood, birth complications, residence | Poor sleep (CBCL) | 5–6 yrs | Maternal depression linked to more poor sleep; happiness during pregnancy was protective. Child behaviour mediated effects. |
| (McDonald et al., 2014) | Longitudinal (twins) | Ethnicity, maternal education, TV use, birth weight | Night sleep duration, night waking | 0–16 mo | Short sleep associated with low maternal education, minority status, male gender, low birth weight, and evening TV. Late bedtime was a key factor. |
| (Miller and Lumeng, 2018) | Review | SES, family conflict, maternal mental health | Sleep duration, quality | Early childhood | Low income and chaotic environments linked to poor sleep. Maternal mental health was a key factor. |
| (Brambilla et al., 2017) | Cross-sectional | Family structure, screen use, bedtime routines | Sleep duration, awakenings, bedtime | 1–14 yrs | Screen use negatively affected sleep. Early poor sleep predicted later issues. High maternal education was protective. |
| (Smolensky et al., 2015) | Review | Light exposure (ALAN), melatonin suppression | Sleep duration, phasing | Children/adolescents | ALAN before bed reduced sleep and melatonin; children more sensitive than adults. |
| (Singh and Kenney, 2013) | Panel data | Socioeconomic status, screen time, environment | Sleep adequacy | 6–17 yrs | Poor neighbourhood and high screen time linked to increased poor sleep. |
| (Marie-Mitchell and Cole, 2022) | Cross-sectional | Adverse childhood experiences (ACEs) | Poor sleep (unspecified) | 5–11 yrs | Higher ACE scores strongly associated with poor sleep. |
| (Cain and Gradisar, 2010) | Review | Media use, parental control, environment | Sleep duration, latency, quality | 5–17 yrs | Media use delayed sleep and disrupted circadian rhythm via arousal and light exposure. |
| (Dube et al., 2017) | Cross-sectional | EECD use, reading habits | Sleep duration, quality, efficiency | 10–11 yrs | EECD use before bed linked to shorter, poorer quality sleep. |
| (Lercher et al., 2013) | Cross-sectional | Traffic noise | Sleep disturbance | 8–11 yrs | Perceived noise, not measured exposure, linked to sleep disturbance. |
| (Nuutinen et al., 2013) | Cohort | Media use, family structure | Sleep duration, bedtime patterns | 10–11 yrs | Media use predicted later bedtimes and irregular sleep. Bedroom media presence linked to poor habits. |
| (Gariepy et al., 2020) | Cross-sectional | SES, gender | Sleep duration, social jetlag | 11–15 yrs | Sleep patterns varied by country but not clearly by SES and gender across countries. Older adolescents reported shorter sleep duration. |

Table S4. Summary of the studies on partial links sleep to mental health and cognition outcomes.

| **Study** |  | **Sleep** | **Outcome** | **Age** | **Findings** |
| --- | --- | --- | --- | --- | --- |
| (Williamson et al., 2020) | Longitudinal  Follow up every 2 yrs until the age 10-11 yrs. | Sleep problem trajectories Difficulty getting asleep Not happy to sleep alone Waking during the night | Self-control  Social Skills Improvement Rating System Quality of life  Cognitive and academic skills Internalizing and externalizing concerns | 0–1 yrs. | Persistent poor sleep was linked to the most severe impairments across outcomes (except perceptual reasoning). Middle-childhood onset was associated with emotional/behavioural issues and lower quality of life. Even mild or early sleep issues showed elevated internalizing symptoms and reduced caregiver-reported quality of life, though to a lesser extent. |
| (Lam and Chung, 2017) | Cross-sectional | Children's sleep habit (Questionnaire)  Bedtime resistance Parasomnia  breathing disorder | Externalizing behaviours | Age range: 2.33–7.17 yrs | In low-SES families, all poor sleep were linked to externalizing behaviours, with bedtime resistance also associated with poorer pre-academic performance. In high-SES families, parasomnia and disordered breathing were modestly linked to externalizing behaviours. |
| (Paruthi et al., 2016) | Review | Nighttime sleep duration | Attention behaviour and learning difficulties Academic performance Depression Self-harm Suicidal thoughts and attempts | 0-18 yrs. | Short sleep duration across infancy to adolescence was linked to poorer mental health (e.g., emotional dysregulation, depression, self-harm), lower cognitive performance, and developmental delays. Effects vary by age, with a U-shaped relationship in adolescents—both too little and too much sleep associated with negative outcomes. Early short sleep predicted behavioural issues and reduced language and problem-solving skills. Sleep restriction in school-aged children impaired memory, attention, and academic performance. |
| (Morales-Muñoz et al., 2020) | Longitudinal  6mo – 13yrs | Night awakening  Irregular sleep Nightmares Bedtime Nighttime sleep duration | Psychotic experiences Borderline personality disorder symptoms | 6mo,18mo, 30mo, 3,5y, 4,8y, and 5,8y   Psychotic experience at 12-13 yrs. | Frequent night awakenings at 18 months and irregular sleep at 6, 30, and 70 months were linked to later psychotic experiences. Shorter sleep duration and later bedtimes at 3.5 years were associated with borderline personality symptoms. Depression at age 10 mediated the relationship between early sleep disturbances and adolescent psychosis. |
| (Meltzer and Mindell, 2006) | Review | Nighttime sleep duration Sleep difficulties | ADHD ASD Anxiety Attention  Learning Behaviour | 0-18yrs | Poor sleep was closely linked to ADHD, depression, and anxiety, with evidence of a bidirectional relationship—treating sleep issues can alleviate ADHD symptoms. In children with ASD, causes of sleep disturbances may include melatonin dysregulation, anxiety, or neurological factors. Developmentally, poor sleep among toddlers (12–36 months) was associated with worse daytime behaviour; in preschoolers (4–5 years), sleep issues may become chronic. For school-aged children (6–12 years), sleep restriction impaired attention, memory, and learning. In adolescents, sleep deprivation negatively affected mood, cognition, and academic performance. |
| (Touchette et al., 2007) | Longitudinal | Nighttime sleep duration Daytime sleepiness | Externalizing problems, (hyperactivity impulsivity) Inattention Non-verbal skills | 2.5 – 6 yrs | Among four identified sleep trajectories, children with persistently short sleep (<10 hours), especially before age 3.5, showed higher levels of hyperactivity and lower cognitive performance on neurodevelopmental assessments. |
| (Gregory and Sadeh, 2012) | Review | Dyssomnia Parasomnias | Combined anxiety/ depression Anxiety ADHD Aggression, conduct disorder and addiction ASD Pervasive developmental disorders | Childhood and adolescence | Sleep disturbances were linked to anxiety and depression, with stronger associations as children age. These issues may predict later mental health problems, though findings vary. Mechanisms include neurobiological (e.g., serotonin, melatonin, cortisol), psychosocial (e.g., family dysfunction, bullying, SES), and emotional regulation pathways. Sleep-deprived individuals show heightened amygdala reactivity and reduced prefrontal control, impairing emotional regulation. |
| (Chaput et al., 2016) | Review | Nighttime sleep duration | Emotional regulation Quality of life/well-being | 5- 17 yrs. | Shorter sleep duration was consistently linked to poorer emotional regulation and increased screen time. It was also associated with impaired growth. However, evidence for effects on cognitive and motor development, physical activity, and overall well-being was inconsistent. |
| (Wang et al., 2016) | Longitudinal | Sleep behaviour | Aggressive behaviour Attention problems Anxiety Depression | 5-17 yrs. | While poor sleep generally declined with age, children with worsening sleep patterns over time (“troubled sleepers”) showed increased aggression and attention issues by age 17. The relationship between sleep and behaviour was bidirectional. |
| (Macchitella et al., 2020) | Cross-sectional | Sleep restoration Paediatric Daytime Sleepiness Scales | Neuropsychological assessment Reading and Comprehension  Spelling Mathematical  comprehension Handwriting speed Visuospatial memory Sustained and selective attention Visio-Spatial | 7.8 to 11.2 yrs. | Sleepiness in children was linked specifically to reduced performance in verbal and complex cognitive tasks (e.g., reading, comprehension, math), but not in visuospatial abilities—suggesting it primarily affects higher-order verbal functions. |
| (Goldstone et al., 2020) | Longitudinal  1yr follow up | Disorders of initiating and maintaining sleep Sleep-wake transition disorders Sleep restoration excessive somnolence Nighttime sleep duration | Internalizing and externalizing symptoms Depression | 9-10 yrs. | Baseline sleep disturbances predicted higher internalizing, externalizing, and depression scores. The effect was stronger in girls, indicating a sex-specific vulnerability. Sleep disturbance was a more reliable predictor of mental health outcomes than sleep duration. |
| (Roberts et al., 2009) | Longitudinal  12mo follow up | Nighttime sleep duration Insomnia | Depression Self-esteem Perceived mental health Problems at school Life satisfaction Academic functioning Grades | 11-17 yrs | Sleep duration under 6 hours linked to increased risk of depression and poorer academic performance. Insomnia further predicted school problems, low life satisfaction, poor mental health, and lower grades. |
| (Chatburn et al., 2013) | Cross-sectional | Poor sleep | Behavioural problems assessed by parents  Resilience self-reports | 7-18 yrs. (mean 11.25) | Poor sleep was strongly correlated with lower resilience. Resilience acted as a mediator between sleep disturbances and internalizing/externalizing behavioural issues, including depression and anxiety. |
| (Kortesoja et al., 2020) | Longitudinal study  2-yrs follow-up | Nighttime sleep duration  Poor sleep | Total difficulties on psychosocial behaviour Emotional symptoms Conduct problems Hyperactivity  Peer problems | Baseline 12–13 yrs. Follow-up 1: 15–16 yrs. Follow-up 2: 17–18 yrs. | Short sleep duration during adolescence linked to increased emotional symptoms, conduct problems, and hyperactivity. These psychosocial difficulties contributed to worsening sleep later in adolescence, indicating a bidirectional relationship. |
| (Raniti et al., 2017) | Cross-sectional | Sleep behaviour Nighttime sleep duration Sleep restoration Subjective sleep quality  Sleep onset latency  Habitual sleep efficiency  Sleep disturbances  Use of sleeping medication Daytime dysfunction. | Depressive symptoms Somatic complaints Motor activity Interpersonal relations | Mean 15.8 yrs Range 11.9–19.7 | Short sleep duration and poor subjective sleep quality partially mediated the link between age and depressive symptoms, though not sleep disturbance, efficiency, or latency. Conversely, depressive symptoms also mediated the relationship between age and sleep quality, suggesting that rising adolescent depression may be partly driven by sleep related developmental changes. |
| (El-Sheikh et al., 2020) | Cross-sectional | Nighttime sleep duration Sleep restoration Sleep efficiency Sleep quality | Internalizing symptoms Externalizing symptoms Cognitive functioning | Mean age = 17.3 yrs (SD = 9.12 mo.) | Shorter, less efficient, and subjectively poor sleep linked to increased internalizing and externalizing symptoms and reduced cognitive performance. These associations were stronger among adolescents from lower socioeconomic backgrounds. |
| (Hysing et al., 2022) | Cross-sectional | Insomnia (DSM-5) Sleep behaviour Delayed sleep-wake-phase disorder Wake-after-sleep onset  Nighttime sleep duration Time in bed Sleep-onset latency  Sleep restoration Sleep efficiency | Depression Anxiety ADHD Conduct disorder Trauma-related disorder Autism Eating disorder Psychotic disorder | 17-19 yrs | Poor sleep contributed to and resulted from psychiatric disorders. Insomnia was significantly more prevalent among adolescents with ADHD (28.7%) and depression (48%) compared to peers (16.7%). Those with psychiatric diagnoses had shorter sleep duration, longer sleep-onset latency and wake-after-sleep onset, and lower sleep efficiency—except in cases of psychosis. |
| (Clinkinbeard et al., 2011) | Cross-sectional | Nighttime sleep duration,  Self-reported | Externalizing behaviours -Property delinquency  -Violent delinquency | 13-19 yrs (mean 15.7) | Fewer hours of sleep were associated with more delinquency (both property and violent). |
| (Jamieson et al., 2020) | Review | Sleep duration Sleep deprivation | Mental ill health | 14-18 yrs | Increased sleep deprivation during adolescence is linked to higher rates of mental disorders. This relationship may be mediated by delayed brain maturation—specifically, underdevelopment of the uncinate fasciculus—reducing emotional regulation through impaired top-down control. |
| (Xu et al., 2020) | Longitudinal  Follow up every 3 mo. for 4 times | Sleep disturbance | Depressive symptoms | 15–24 yrs | While the direct effect of sleep disturbance on depressive symptoms was not significant, indirect effects through emotional exhaustion and sleep-related worry were. These mediators highlight the psychological pathways linking sleep issues to depression. |
| (Tavernier and Willoughby, 2015) | Longitudinal  1 baseline + 2 follow-ups. | Sleep behaviour Difficulty falling asleep Difficulty staying asleep Waking up too early Sleep restoration Problem staying awake Daytime fatigue,  Inability to function  Nighttime sleep duration The difference in average bedtimes between the week and the weekend Weekend oversleep | Social relations Meeting people and making friends  Have several close social ties  Satisfied with participation in social activities  Emotion Regulation | 17-25 yrs | Fewer poor sleep associated with stronger social ties, mediated by better emotion regulation. Conversely, positive social relationships also predicted fewer sleep issues through the same regulatory pathway. |

## Figure S 1


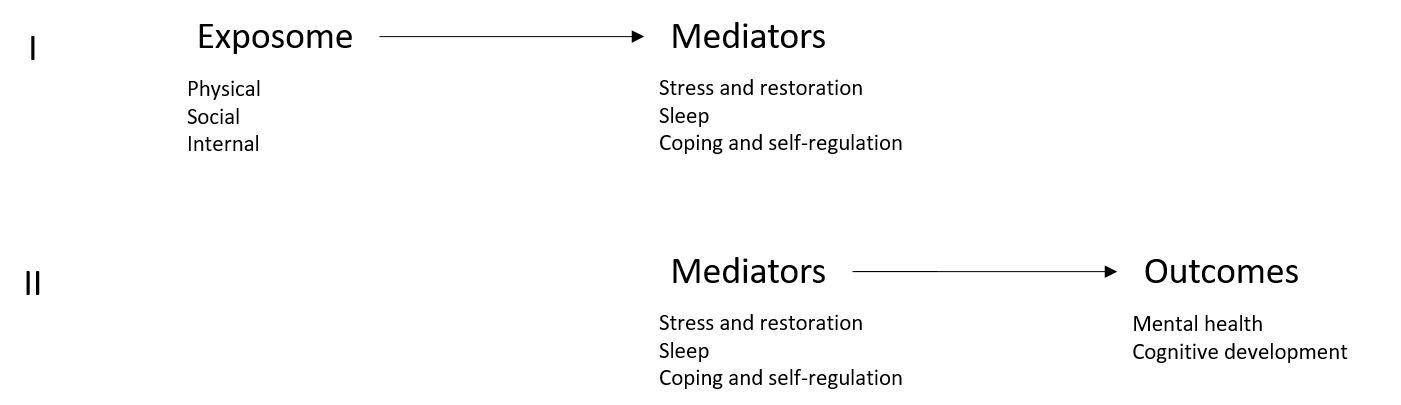


Figure S1. Partial pathways explored for mechanistic evidence

## Supplement S1.

Search string used in the updated searches in Scopus (December 2022, December 2023, January 2025) - limited to: keywords human, humans, publication year 2020-2023, language English, publication type article/review

( TITLE-ABS-KEY ( sleep* OR "sleep duration" OR insomnia OR parasomnia* OR nightmares OR "night terrors" OR dyssomnia* OR rem OR non-rem OR polysomnogr* OR awakening OR arousal OR "circadian rhythm" OR "bedtime resistance" OR wake OR wakening* OR nocturnal OR overnight OR evening OR morning OR tiredness OR fatigue OR "daytime functioning" ) ) AND ( ( ( TITLE-ABS-KEY ( neonat* OR zygot* OR embryo* OR fetus OR foetus OR fetal OR newborn* OR child* OR infant OR infancy OR toddler OR puberty OR pubertal OR "early life" OR teen* OR youth* OR adolescen* OR juvenile* OR young* OR pre-teen* OR preteen* OR pre-adolescen* OR preadolescen* OR pediatric OR student OR pre-school OR school* ) ) AND ( ( TITLE-ABS-KEY ( exposome OR "Lifecourse epidemiology" OR co-expos* OR omics ) ) OR ( TITLE-ABS-KEY ( expos* OR environment* OR hazards OR risk OR risks ) W/4 TITLE-ABS-KEY ( joint OR combi* OR complex OR synerg* OR additive* OR multi OR multiple OR cumulat* OR collect* OR interact* OR compensat* OR lifetime OR life-long OR totality OR several OR interplay OR holistic OR "more than one" OR comprehensive OR stress* OR overall ) ) ) AND ( TITLE-ABS-KEY ( nois* OR pollut* OR "particulate matter" OR dust OR vibrat* OR electromagn* OR climate OR weather OR temperature OR humid* OR season* OR water OR food OR nutri* OR diet OR green OR blue OR natur* OR outdoor OR restorative OR mould OR mold OR damp* OR "built environment" OR indoor OR housing OR dwelling OR residential OR urban OR rural OR school OR pre-school OR daycare OR kindergarten ) ) AND ( ( TITLE-ABS-KEY ( mental OR psych* OR internali* OR externali* OR "conduct disorder" OR emotion* OR "affective symptoms" OR mood OR temper* OR sadness OR anxiety OR depress* OR neurodevelop* OR behaviour* OR behavior* OR neuropsychology OR neurobehavior OR neurobehaviour OR autis* OR personality OR obsessive OR "eating disorder" OR hyperactiv* OR "oppositional defiant" OR p-factor OR tics OR self-harm OR cognit* OR reading OR spelling OR math* OR arithmetic* OR literacy OR intellect* OR intelligen* OR iq OR language OR speech OR learn* OR memory OR "executive functions" OR "executive function" OR "executive functioning" OR attenti* OR inattenti* OR competence* OR skill* OR "problem solving" OR well-being OR well-being OR "well being" OR "quality of life" OR "peer-relation" OR "approval seeking" OR empath* OR empower* OR agressi* OR anger OR irritation OR helpless* OR hopeless* OR attachment OR insecur* OR secur* OR powerless* OR addiction OR addicted OR delinquen* OR prosocial OR pro-social OR stubborn OR tantrum OR antisocial OR sdq ) ) OR ( TITLE-ABS-KEY ( school OR academic OR educat* ) W/4 TITLE-ABS-KEY ( achievement OR attainment OR perform* OR grades ) ) ) ) AND ( TITLE-ABS-KEY ( mediat* OR pathway* OR mechanis* OR "structural equation" OR "path analysis" OR moderat* ) ) )

## Supplement S2

Search string used in the update searches in PsycInfo (December 2022, December 2023, January 2025) *- Limiters Publication year 2020-2023. Language English, Peer reviewed*

((ab(neonat* OR zygot* OR embryo* OR fetus OR foetus OR fetal OR newborn* OR child* OR infant OR infancy OR toddler OR puberty OR pubertal OR "early life" OR teen* OR youth* OR adolescen* OR juvenile* OR young* OR pre-teen* OR preteen* OR pre-adolescen* OR preadolescen* OR pediatric OR student OR pre-scool OR school) OR ti(neonat* OR zygot* OR embryo* OR fetus OR foetus OR fetal OR newborn* OR child* OR infant OR infancy OR toddler OR puberty OR pubertal OR "early life" OR teen* OR youth* OR adolescen* OR juvenile* OR young* OR pre-teen* OR preteen* OR pre-adolescen* OR preadolescen* OR pediatric OR student OR pre-scool OR school)) AND ((ti(school OR academic OR educat*) NEAR/4 ti(achievement OR attainment OR perform* OR grades) OR ab(school OR academic OR educat*) NEAR/4 ab(achievement OR attainment OR perform* OR grades)) OR (ab(mental OR psych* OR internali* OR externali* OR "conduct disorder" OR emotion* OR "affective symptoms" OR mood OR temper* OR Sadness OR anxiety OR depress* OR neurodevelop* OR behavior* OR behavior* OR neuropsychology OR neurobehavior OR neurobehaviour OR spelling OR math* or arithmetic* OR literacy* OR memory OR ”executive functions” or ”executive function” or ”executive functioning” OR attenti* OR inattenti* OR skill* OR irritation OR SDQ OR hyperactiv* OR autis* OR personality OR obsessive OR "eating disorder" OR hyperactiv* OR "oppositional defiant" OR p-factor OR tics OR self-harm OR Cognit* OR reading OR intellect* OR intelligen* OR iq OR language OR speech OR learn* OR competence* OR "problem solving" OR well-being OR well-being OR "well being" OR "quality of life" OR "peer-relation" OR "approval seeking" OR empath* OR empower* OR agressi* OR anger OR helpless* OR hopeless* OR attachment OR insecur* OR secur* OR powerless* OR addiction OR addicted OR delinquen* OR prosocial OR pro-social OR stubborn OR tantrum OR antisocial) OR ti(mental OR psych* OR internali* OR externali* OR "conduct disorder" OR emotion* OR "affective symptoms" OR mood OR temper* OR Sadness OR anxiety OR depress* OR neurodevelop* OR behavior* OR behavior* OR neuropsychology OR neurobehavior OR neurobehaviour OR spelling OR math* or arithmetic* OR literacy* OR memory OR ”executive functions” or ”executive function” or ”executive functioning” OR attenti* OR inattenti* OR skill* OR irritation OR SDQ OR hyperactiv* OR autis* OR personality OR obsessive OR "eating disorder" OR hyperactiv* OR "oppositional defiant" OR p-factor OR tics OR self-harm OR Cognit* OR reading OR intellect* OR intelligen* OR iq OR language OR speech OR learn* OR competence* OR "problem solving" OR well-being OR well-being OR "well being" OR "quality of life" OR "peer-relation" OR "approval seeking" OR empath* OR empower* OR agressi* OR anger OR helpless* OR hopeless* OR attachment OR insecur* OR secur* OR powerless* OR addiction OR addicted OR delinquen* OR prosocial OR pro-social OR stubborn OR tantrum OR antisocial))) AND (ab(Nois* OR Pollut* OR "particulate matter" OR dust OR Vibrat* OR Electromagn* OR climate OR weather OR temperature OR Humid* OR Season* OR water OR food OR nutri* OR diet OR green OR blue OR natur* OR outdoor OR restorative OR Mould OR mold OR Damp* OR "built environment" OR indoor OR housing OR dwelling OR residential OR urban OR rural OR school OR pre-school OR daycare OR kindergarten) OR ti(Nois* OR Pollut* OR "particulate matter" OR dust OR Vibrat* OR Electromagn* OR climate OR weather OR temperature OR Humid* OR Season* OR water OR food OR nutri* OR diet OR green OR blue OR natur* OR outdoor OR restorative OR Mould OR mold OR Damp* OR "built environment" OR indoor OR housing OR dwelling OR residential OR urban OR rural OR school OR pre-school OR daycare OR kindergarten)) AND ((ab("Lifecourse epidemiology" OR Co-expos* OR omics) OR ti("Lifecourse epidemiology" OR Co-expos* OR omics)) OR (ab(expos* OR environment* OR hazards OR risk OR risks) NEAR/4 ab(joint OR combi* OR complex OR synerg* OR additive* OR multi OR multiple OR cumulat* OR collect* OR interact* OR compensat* OR lifetime OR Life-long OR totality OR several OR interplay OR holistic OR "more than one" OR comprehensive OR stress* OR overall)) OR (ti(expos* OR environment* OR hazards OR risk OR risks) NEAR/4 ti(joint OR combi* OR complex OR synerg* OR additive* OR multi OR multiple OR cumulat* OR collect* OR interact* OR compensat* OR lifetime OR Life-long OR totality OR several OR interplay OR holistic OR "more than one" OR comprehensive OR stress* OR overall))) AND (ab(mediat* OR pathway* OR mechanis* OR "structural equation" OR "path analysis" OR moderat*) OR ti(mediat* OR pathway* OR mechanis* OR "structural equation" OR "path analysis" OR moderat*))) AND (ti(sleep* OR "sleep duration" OR insomnia OR parasomnia* OR nightmares OR "night terrors" OR dyssomnia* OR REM OR non-REM OR polysomnogr* OR awakening OR arousal OR "circadian rhythm" OR “bedtime resistance” OR wake OR wakening* OR nocturnal OR overnight OR evening OR morning OR tiredness OR fatigue OR “daytime functioning”) OR ab(sleep* OR "sleep duration" OR insomnia OR parasomnia* OR nightmares OR "night terrors" OR dyssomnia* OR REM OR non-REM OR polysomnogr* OR awakening OR arousal OR "circadian rhythm" OR “bedtime resistance” OR wake OR wakening* OR nocturnal OR overnight OR evening OR morning OR tiredness OR fatigue OR “daytime functioning”))
